# Detection of the Onset of the Epidemic Period of Respiratory Syncytial Virus Infection in Japan

**DOI:** 10.3389/fpubh.2019.00039

**Published:** 2019-03-07

**Authors:** Hidetomi Yamagami, Hirokazu Kimura, Takafumi Hashimoto, Isao Kusakawa, Satoshi Kusuda

**Affiliations:** ^1^Medical Affairs, AbbVie GK, Tokyo, Japan; ^2^Department of Health Science, Gunma Paz University Graduate School of Health Science, Gunma, Japan; ^3^Data Science and Stat, AbbVie GK, Tokyo, Japan; ^4^Department of Pediatrics, St. Luke's International Hospital, Tokyo, Japan; ^5^Department of Pediatrics, Faculty of Medicine, Kyorin University, Tokyo, Japan

**Keywords:** respiratory syncytial virus infection, palivizumab, IDWR, epidemic period, the onset of RSV season

## Abstract

Respiratory syncytial virus (RSV) is a leading cause of lower respiratory tract infection in young children worldwide. An annual epidemic of RSV infection generally begins around autumn, reaching a peak at the end of year in Japan, but in 2017 it started in early July and peaked in September. As the onset timing of RSV season varies, it is important to detect the beginning of an epidemic, to enable the implementation of preventive measures. However, there are currently no specified criteria or methods to determine the onset of RSV season in a timely manner. Therefore, we developed a model to detect the epidemic onset, based on data from the Infectious Diseases Weekly Report from 2012 to 2017. The 47 prefectures of Japan span 11 climate zones, which affect the timing of epidemic onset. Therefore, the onset of RSV season was assessed separately in each prefecture. Non-linear regression analysis was performed to generate a mathematical model of the annual epidemic cycle for each prefecture. A search index was used to determine the onset of RSV season, which was estimated using the number of RSV reports per week within an epidemic period (RSV-reports/w) and the number of reported cases included within an epidemic period relative to the total number of RSV reports (capture rate). A number of RSV-reports/w, which was used as a threshold (a number at onset line) to determine the condition of the onset of RSV season, was then estimated based on the search index. The mean number at the onset of RSV season for 47 prefectures was 29.7 reports/week (median 21.0, range 6.0–121.0 reports/ week). The model also showed that the onset of RSV season in 2017 was more than 1 month earlier than the previous year. In conclusion, the model detected epidemic cycles and their onset conditions in all prefectures, despite the 11 climate zones of Japan. The results are expected to contribute to infant medical care by allowing medical personnel to take preventive measures promptly at the beginning of the epidemic RSV season.

## Introduction

Respiratory syncytial virus (RSV) is a major cause of lower respiratory tract infection in infants, and more than 50% of infants are infected for the first time by the age of 1 year, and almost 100% of infants by the age of 2 years (1, 2). Lifelong immunity is not acquired after the primary infection; therefore, reinfection with RSV is universally observed in nearly 50% of infants by the age of 2 years (1). In infants, about 50% of cases of pneumonia, and around 50% to 90% of cases of bronchiolitis are attributed to RSV infection (3). In particular, in premature infants and infants with chronic respiratory diseases such as bronchopulmonary dysplasia, RSV infection tends to be severe (4–7). Also, RSV infection tends to be severe even in infants with congenital heart diseases (8, 9)^1^ and infants with immunodeficiency or Down's syndrome (10). It is important to take preventive measures for these high-risk infants, as RSV infection may take a fatal course in some cases (11).

Because the medicine for RSV infection has not been developed, patients receive supportive treatment mainly by transfusion and respiratory management. In Japan, public health insurance coverage was approved in 2002 for palivizumab (product name: Synagis®), an anti-RSV humanized monoclonal antibody, which is used as a prophylactic medicine to prevent severe lower respiratory tract disease associated with RSV infection in high-risk infants. As palivizumab needs to be administered once a month throughout the epidemic period of RSV infection, for its appropriate use, it is important to accurately detect the onset of the epidemic period.

**Figure 1:**
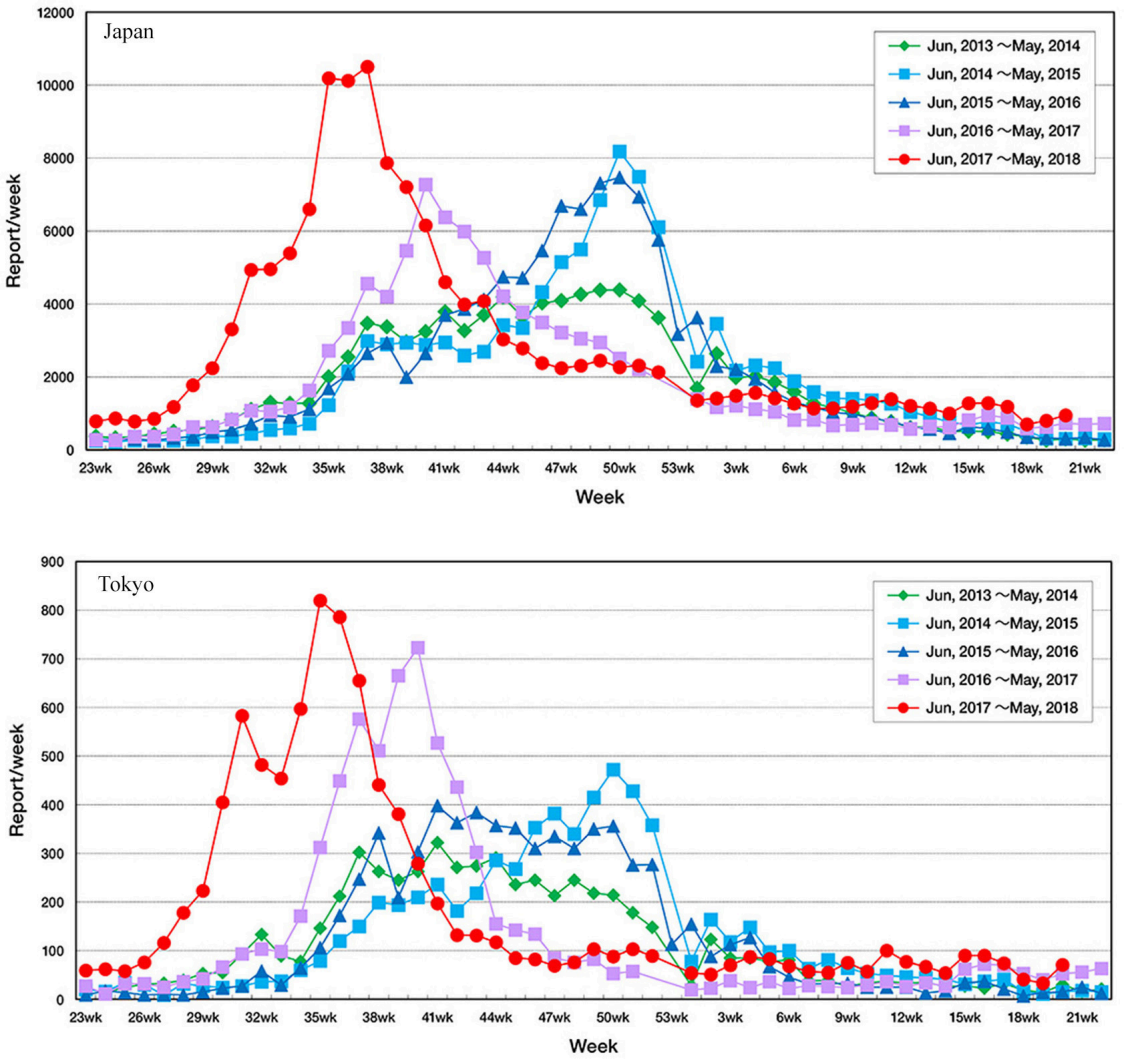
Fluctuation in the number of reported cases of respiratory syncytial virus infection throughout Japan according to the Infectious Diseases Weekly Report nationwide surveillance data in Tokyo and in Japan.

RSV infection is monitored via pediatric sentinel sites under the Act on Prevention of Infectious Diseases and Medical Care for Patients Suffering from Infectious Diseases (11). In this program, physicians at designated notification facilities (~3,000 pediatric medical institutions at fixed points all around Japan to cover 10% of pediatric institutions in Japan) are required to report patients diagnosed with RSV infection based on their symptoms and findings of RSV antigen or by PCR, and the information is published in the Infectious Diseases Weekly Report (IDWR) as national surveillance data by the National Institute of Infectious Diseases (NIID)^1^. In Japan, the epidemic of RSV infection had generally begun around autumn, and the number of reported cases had increased rapidly in autumn, reached a peak at the end of the year, and continued until spring. No significant fluctuations were observed in the epidemic tendency nationwide until 2015; however, the number of reported cases increased from early August in 2016. In 2017, the number of reported cases increased rapidly from early July and reached a peak in September. Tendencies of fluctuations in the onset of the epidemic period are evident, as the number of RSV reports started to increase earlier than in the past several seasons (Figure 1). To resolve the issue, the guideline for the use of palivizumab in Japan was immediately revised in accordance with the observed epidemical change of RSV. As the onset of RSV season changes every year and there are no specified decision criteria for the onset of the epidemic RSV season, the onset of the RSV season should be determined on the basis of the IDWR and other included reports^2^.

In response to this, we investigated the onset of the epidemic period of RSV infection based on the IDWR surveillance data using a newly developed algorithm. The main objective of this study was to detect the onset weeks of the annual RSV epidemics in Japan to enable timely access to preventive measures for severe RSV disease.

## Methods

### Data Source

Six epidemic periods with a 1-year cycle of RSV infection for 46 prefectures and five epidemic periods with a 1-year cycle of RSV infection for Okinawa were analyzed using the IDWR surveillance data from the first week of 2012 to the 24th week of 2018^3^. The numbers of sentinel medical institutions (SMI) in each prefecture were used on the basis of the average numbers of sentinel medical institutions in each prefecture in 2016. Weeks ending logs were referenced from the NIID report^4^.

### Modeling the RSV Season as a Periodic Oscillation

RSV incidence is reported weekly in each prefecture in Japan. Since epidemic cycles vary among regions in Japan, we generated a mathematical model of the annual epidemic cycle for each prefecture by applying non-linear regression analysis to data reported from the first week of 2012 to the 24th week of 2018. The following sine function was used to model the cyclical nature of the annual epidemics, where “Y” represents the number of RSV reports each week, “w” represents the week of the year (1–52) in which the cases were reported, and a_0_, a_1_, a_2_, and a_3_ represent fixed parameters.

(1) $$\begin{array}{r} \text{Y}={\text{a}}_{0}+\left({\text{a}}_{1}\times \text{sin}\left({\text{a}}_{2}+a_{3}\times \left(\text{w}\times \;\pi /180\right)\right)\right). \end{array}$$

Because we focused on the rhythm of the epidemics, year-to-year variations in RSV reporting numbers (the amplitude of the function) were not incorporated into the model, and an upper limit was applied to variable Y. The model parameters were calculated using the “NLIN” non-linear regression procedure in SAS software (version 9.4; SAS Institute, Cary, NC, USA), with Gauss-Newton iteration. We confirmed that the convergence criterion was met for each prefecture. Also, visual confirmation of graphs of each data set confirmed that the incidence of RSV cases in each prefecture of Japan had sinusoidal periodicity.

When there was more than one peak between the 1st week and the 52nd week in a plot, the peak corresponding to the highest reported number of cases was selected as the main peak for calculation purposes. When there was more than one trough between the 1st week and the 52nd week in a plot, the trough falling just before the main peak was selected as the central trough for calculation purposes. The range of a 1-year cycle based on the sinusoidal curve was identified between one trough and next trough. The range of data used to identify epidemic starting points was a 6-year cycle for 46 prefectures, and a 5-year cycle for 1 prefecture (Okinawa). An epidemic period was defined as the period between the onset and the next trough.

The validity of the model was confirmed by determining the proportion of the number of RSV reports falling below the onset line (horizontal line of RSV reporting number at onset) of the RSV season and within the bounds of the sine function.

### Determining the Onset of RSV Season

The main objective of this study was to determine the number of RSV reports at the onset of RSV season in Japan to enable timely access to preventive treatment. Ideally, the determination of period for the epidemic should include as many patients as possible. However, the higher number of RSV reports included, the longer the specified epidemic period becomes. From a health economics perspective, more information about a short epidemic period with more RSV reports is desirable. Accordingly, both the number of RSV reports included within epidemic periods relative to the total number of RSV reports (the capture rate) and the number of RSV reports per week within an epidemic period (RSV-reports/w) must be taken into account when determining epidemic starting points. The search index for both the capture rate and RSV-reports/w was then calculated to determine the onset of the epidemic cycle (see the formula below).

The search index was expressed by the following equation:

$$\begin{array}{l} A\left(x\right)=\frac{\Sigma_{i=1}^{s}\Sigma_{j=onset{\left(x\right)}_{i}}^{Through_{i}}k_{j}}{\Sigma_{i=1}^{s}\left(Trough_{i}-onset{\left(x\right)}_{i}+1\right)}\\ B\left(x\right)=\frac{\Sigma_{i=1}^{s}\Sigma_{j=onset{\left(x\right)}_{i}}^{Trough_{i}}k_{j}}{\Sigma_{j=Trough\left(0\right)}^{Trough\left(s\right)}k_{j}}\\ INDEX\left(x\right)=\frac{A\left(x\right)}{max\left\{x|A\left(x\right)\right\}}+\frac{B\left(x\right)}{max\left\{x|B\left(x\right)\right\}} \end{array}$$

*A(x)* denotes RSV-reports/w (the number of RSV reported per week)

*B(x)* denotes capture rate (the number of RSV reports included within onset—trough periods relative to the total number of RSV reports in the dataset).

*INDEX(x)* denotes the search index. The capture rate (A(x)) and RSV- reports/w (B(x)) were converted into the same scale and summed.

The other variables were as follows:

■ *x*, a percentile value ranging from 0 to 100 in integer units;
■ *s*, the number of epidemic cycles in the dataset;
■ *Onset(x)*, the ordinal number of each onset -week^*^ within the dataset from 1 to *x* in integer units;
■ *Trough*, the ordinal number of each trough-week within the dataset from 0 to *x* in integer units;
■ *k*, the number of RSV reports in a given week.

^*^The procedure to set the onset was as follows: The number of RSV reports for each prefecture was transposed into percentile rank, and the minimum percentile rank that was greater than the percentile value was selected. For the 101 percentile values, 101 percentile ranks were selected for each prefecture. The reporting week corresponding to the intersection of these percentile ranks and the increasing slope of the sinusoidal curve was taken as the onset.

Percentile rank of the highest index was set as the onset line, which provided a number of RSV reports/w and was used as a threshold horizontal line to detect the onset of the RSV season. The onset of each epidemic was identified from the intersection of the onset line of the epidemic and the increasing slope of the sinusoidal curve.

The validity of the determined onset was confirmed by calculating a capture rate.

The analyses were first performed on the data reported in Tokyo, the model was then validated using the data for 47 prefectures.

## Results

### Determination of the Onset of RSV Season in Tokyo

**Figure 2:**
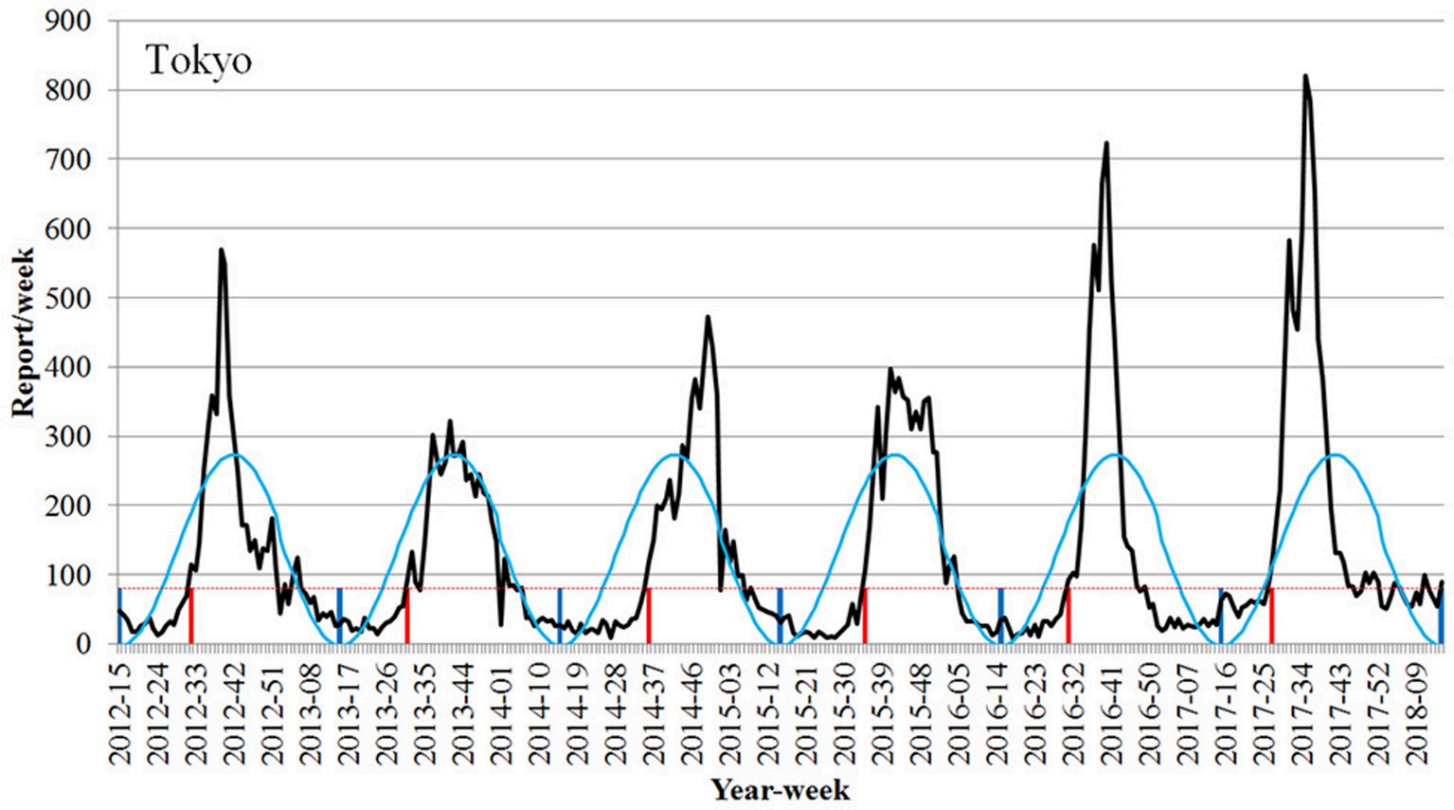
Detection of the onset week of the epidemic season of respiratory syncytial virus infection using the Infectious Diseases Weekly Report nationwide surveillance data in Tokyo (2012–2017 seasons). Black line, number of RSV report; light blue line, RSV epidemic cycle; dotted red line, onset line of RSV season; vertical red line, onset week; vertical blue line, trough of epidemic cycle.

The number of RSV reports/w at the onset of RSV season in Tokyo was 81 reports/week (Figure 2). When 81 reports/week was set as the onset line of the epidemic period in Tokyo, the onset of epidemic periods was detected through all the years from 2012 to 2017.

The onset of the epidemic period was the 32nd week (August 6–12) in 2012, the 31st week (July 29–August 4) in 2013, the 36th week (September 1–7) in 2014, the 35th week (August 24–30) in 2015, and the 31st week (August 1–7) in 2016. The onset was the 27th week (July 3–9) in 2017, which was earlier as compared to those previously observed (Figure 2, Table 1).

**Table 1:** The onset of RSV season in 47 prefectures.

**Prefecture**	**Number at a onset line of RSV season**	**Number of weekly report/SMI**	**Onset week of RSV season**					
			**2012**	**2013**	**2014**	**2015**	**2016**	**2017**
Hokkaido	71	0.50	Week 40 (Oct.1–7)	Week 28 (Jul.8–14)	Week 43 (Oct.20–26)	Week 36 (Aug.31–Sep.6)	Week 29 (Jul.18–24)	Week 21 (May.22–28)
Aomori	20	0.49	Week 31 (Jul.30–Aug.5)	Week 37 (Sep.9–15)	Week 42 (Oct.13–19)	Week 36 (Aug.31–Sep.6)	Week 34 (Aug.22–28)	Week 27 (Jul.3–9)
Iwate	21	0.53	Week 37 (Sep.10–16)	Week 37 (Sep.9–15)	Week 39 (Sep.22–28)	Week 30 (Jul.20–26)	Week 34 (Aug.22–28)	Week 27 (Jul.3–9)
Miyagi	28	0.47	Week 28 (Jul.9–15)	Week 31 (Jul.29–Aug.4)	Week 45 (Nov.3–9)	Week 34 (Aug.17–23)	Week 34 (Aug.22–28)	Week 25 (Jun.19–25)
Akita	11	0.31	Week 30 (Jul.23–29)	Week 36 (Sep.2–8)	Week 39 (Sep.22–28)	Week 39 (Sep.21–27)	Week 37 (Sep.12–18)	Week 31 (Jul.31–Aug.6)
Yamagata	21	0.72	Week 35 (Aug.27–Sep.2)	Week 35 (Aug.26–Sep.1)	Week 39 (Sep.22–28)	Week 36 (Aug.31–Sep.6)	Week 34 (Aug.22–28)	Week 31 (Jul.31–Aug.6)
Fukushima	35	0.76	Week 35 (Aug.27–Sep.2)	Week 32 (Aug.5–11)	Week 43 (Oct.20–26)	Week 35 (Aug.24–30)	Week 34 (Aug.22–28)	Week 24 (Jun.12–18)
Ibaraki	25	0.33	Week 36 (Sep.3–9)	Week 36 (Sep.2–8)	Week 41 (Oct.6–12)	Week 42 (Oct.12–18)	Week 35 (Aug.29–Sep.4)	Week 28 (Jul.10–16)
Tochigi	22	0.46	Week 36 (Sep.3–9)	Week 36 (Sep.2–8)	Week 44 (Oct.27–Nov.2)	Week 40 (Sep.28–Oct.4)	Week 35 (Aug.29–Sep.4)	Week 32 (Aug.7–13)
Gunma	21	0.37	Week 37 (Sep.10–16)	Week 40 (Sep.30–Oct.6)	Week 44 (Oct.27–Nov.2)	Week 41 (Oct.5–11)	Week 33 (Aug.15–21)	Week 31 (Jul.31–Aug.6)
Saitama	56	0.35	Week 35 (Aug.27–Sep.2)	Week 32 (Aug.5– 11)	Week 37 (Sep.8–14)	Week 36 (Aug31–Sep.6)	Week 34 (Aug.22–28)	Week 27 (Jul.3–9)
Chiba	35	0.26	Week 35 (Aug.27–Sep.2)	Week 30 (Jul.22–28)	Week 36 (Sep.1–7)	Week 35 (Aug.24–30)	Week 34 (Aug.22–28)	Week 29 (Jul.17–23)
Tokyo	81	0.31	Week 32 (Aug.6–12)	Week 31 (Jul.29–Aug.4)	Week 36 (Sep.1–7)	Week 35 (Aug.24–30)	Week 31 (Aug.1–7)	Week 27 (Jul.3–9)
Kanagawa	47	0.22	Week 36 (Sep.3–9)	Week 32 (Aug.5–11)	Week 36 (Sep.1–7)	Week 35 (Aug.24–30)	Week 31 (Aug.1–7)	Week 24 (Jun.12–18)
Niigata	43	0.74	Week 39 (Sep.24–30)	Week 34 (Aug.19–25)	Week 36 (Sep.1–7)	Week 34 (Aug.17–23)	Week 33 (Aug.15–21)	Week 24 (Jun.12–18)
Toyama	17	0.61	Week 36 (Sep.3–9)	Week 37 (Sep.9–15)	Week 39 (Sep.22–28)	Week 41 (Oct.5–11)	Week 35 (Aug.29–Sep.4)	Week 29 (Jul.17–23)
Ishikawa	17	0.59	Week 35 (Aug.27–Sep.2)	Week 35 (Aug.26–Sep.1)	Week 40 (Sep.29–Oct.5)	Week 37 (Sep.7–13)	Week 35 (Aug.29–Sep.4)	Week 31 (Jul.31–Aug.6)
Fukui	15	0.68	Week 36 (Sep.3–9)	Week 32 (Aug.5–11)	Week 35 (Aug.25–31)	Week 40 (Sep.28–Oct.4)	Week 37 (Sep.12–18)	Week 31 (Jul.31–Aug.6)
Yamanashi	6	0.25	Week 40 (Oct.1–7)	Week 38 (Sep.16–22)	Week 37 (Sep.8–14)	Week 41 (Oct.5–11)	Week 36 (Sep.5–11)	Week 33 (Aug.14–20)
Nagano	21	0.40	Week 45 (Nov.5–11)	Week 43 (Oct.21–27)	Week 40 (Sep.29–Oct.5)	Week 41 (Oct.5–11)	Week 39 (Sep.26–Oct.2)	Week 34 (Aug.21–27)
Gifu	16	0.31	Week 38 (Sep.17–23)	Week 33 (Aug.12–18)	Week 36 (Sep.1–7)	Week 38 (Sep.14–20)	Week 36 (Sep.5–11)	Week 31 (Jul.31–Aug.6)
Shizuoka	35	0.39	Week 36 (Sep.3–9)	Week 37 (Sep.9–15)	Week 38 (Sep.15–21)	Week 35 (Aug.24–30)	Week 36 (Sep.5–11)	Week 30 (Jul.24–30)
Aichi	73	0.40	Week 38 (Sep.17–23)	Week 36 (Sep.2–8)	Week 36 (Sep.1–7)	Week 40 (Sep.28–Oct.4)	Week 37 (Sep.12–18)	Week 30 (Jul.24–30)
Mie	29	0.64	Week 38 (Sep.17–23)	Week 40 (Sep.30–Oct.6)	Week 40 (Sep.29–Oct.5)	Week 37 (Sep.7–13)	Week 34 (Aug.22–28)	Week 31 (Jul.31–Aug.6)
Shiga	14	0.45	Week 38 (Sep.17–23)	Week 41 (Oct.7–13)	Week 37 (Sep.8–14)	Week 41 (Oct.5–11)	Week 37 (Sep.12–18)	Week 32 (Aug.7–13)
Kyoto	17	0.23	Week 36 (Sep.3–9)	Week 35 (Aug.26–Sep.1)	Week 36 (Sep.1–7)	Week 37 (Sep.7–13)	Week 35 (Aug.29–Sep.4)	Week 30 (Jul.24–30)
Osaka	121	0.61	Week 36 (Sep.3–9)	Week 35 (Aug.26–Sep.1)	Week 35 (Aug.25–31)	Week 37 (Sep.7–13)	Week 33 (Aug.15–21)	Week 28 (Jul.10–16)
Hyogo	58	0.45	Week 39 (Sep.24–30)	Week 37 (Sep.9–15)	Week 36 (Sep.1–7)	Week 40 (Sep.28–Oct.4)	Week 35 (Aug.29–Sep.4)	Week 31 (Jul.31–Aug.6)
Nara	19	0.56	Week 37 (Sep.10–16)	Week 37 (Sep.9–15)	Week 38 (Sep.15–21)	Week 37 (Sep.7–13)	Week 35 (Aug.29–Sep.4)	Week 30 (Jul.24–30)
Wakayama	15	0.48	Week 38 (Sep.17–23)	Week 36 (Sep.2–8)	Week 33 (Aug.11–17)	Week 42 (Oct.12–18)	Week 36 (Sep.5–11)	Week 27 (Jul.3–9)
Tottori	11	0.58	Week 39 (Sep.24–30)	Week 37 (Sep.9–15)	Week 36 (Sep.1–7)	Week 37 (Sep.7–13)	Week 36 (Sep.5–11)	Week 29 (Jul.17–23)
Shimane	15	0.65	Week 36 (Sep.3–9)	Week 33 (Aug.12–18)	Week 35 (Aug.25–31)	Week 35 (Aug.24–30)	Week 36 (Sep.5–11)	Week 34 (Aug.21–27)
Okayama	15	0.28	Week 37 (Sep.10–16)	Week 37 (Sep.9–15)	Week 37 (Sep.8–14)	Week 35 (Aug.24–30)	Week 36 (Sep.5–11)	Week 33 (Aug.14–20)
Hiroshima	33	0.46	Week 32 (Aug.6–12)	Week 30 (Jul.22–28)	Week 35 (Aug.25–31)	Week 33 (Aug.10–16)	Week 31 (Aug.1–7)	Week 30 (Jul.24–30)
Yamaguchi	29	0.60	Week 35 (Aug.27–Sep.2)	Week 30 (Jul.22–28)	Week 36 (Sep.1–7)	Week 29 (Jul.13–19)	Week 31 (Aug.1–7)	Week 28 (Jul.10–16)
Tokushima	23	1.00	Week 40 (Oct.1–7)	Week 37 (Sep.9–15)	Week 37 (Sep.8–14)	Week 36 (Aug.31–Sep.6)	Week 35 (Aug.29–Sep.4)	Week 31 (Jul.31–Aug.6)
Kagawa	17	0.61	Week 37 (Sep.10–16)	Week 36 (Sep.2–8)	Week 39 (Sep.22–28)	Week 40 (Sep.28–Oct.4)	Week 37 (Sep.12–18)	Week 32 (Aug.7–13)
Ehime	20	0.54	Week 35 (Aug.27–Sep.2)	Week 33 (Aug.12–18)	Week 36 (Sep.1–7)	Week 37 (Sep.7–13)	Week 35 (Aug.29–Sep.4)	Week 29 (Jul.17–23)
Kochi	13	0.43	Week 36 (Sep.3–9)	Week 37 (Sep.9–15)	Week 37 (Sep.8–14)	Week 34 (Aug.17–23)	Week 35 (Aug.29–Sep.4)	Week 31 (Jul.31–Aug.6)
Fukuoka	64	0.53	Week 30 (Jul.23–29)	Week 28 (Jul.8–14)	Week 32 (Aug.4–10)	Week 29 (Jul.13–19)	Week 28 (Jul.11–17)	Week 28 (Jul.10–16)
Saga	16	0.70	Week 35 (Aug.27–Sep.2)	Week 33 (Aug.12–18)	Week 32 (Aug.4–10)	Week 34 (Aug.17–23)	Week 39 (Sep.26–Oct.2)	Week 32 (Aug.7–13)
Nagasaki	17	0.39	Week 35 (Aug.27–Sep.2)	Week 33 (Aug.12–18)	Week 33 (Aug.11–17)	Week 32 (Aug.3–9)	Week 32 (Aug.8–14)	Week 29 (Jul.17–23)
Kumamoto	26	0.53	Week 35 (Aug.27–Sep.2)	Week 34 (Aug.19–25)	Week 36 (Sep.1–7)	Week 35 (Aug.24–30)	Week 30 (Jul.25–31)	Week 30 (Jul.24–30)
Oita	17	0.47	Week 33 (Aug.13–19)	Week 38 (Sep.16–22)	Week 35 (Aug.25–31)	Week 30 (Jul.20–26)	Week 38 (Sep.19–25)	Week 30 (Jul.24–30)
Miyazaki	29	0.81	Week 31 (Jul.30–Aug.5)	Week 31 (Jul.29–Aug.4)	Week 36 (Sep.1–7)	Week 31 (Jul.27–Aug.2)	Week 27 (Jul.4–10)	Week 31 (Jul.31–Aug.6)
Kagoshima	21	0.38	Week 27 (Jul.2–8)	Week 26 (Jun.24–30)	Week 33 (Aug.11–17)	Week 23 (Jun.1–7)	Week 24 (Jun.13–19)	Week 26 (Jun.26–Jul.2)
Okinawa	17	0.50	-	Week 7 (Feb.11–17)	Week 8 (Feb.17–23)	Week 8 (Feb.16–22)	Week 4 (Jan.25–31)	Week 5 (Jan. 30–Feb.5)

*RSV, respiratory syncytial virus*.

*number at an onset line of RSV season was used as a threshold to determine an onset of RSV season*.

When converting the value to the number of cases reported per SMI with the view to generalize the findings to other prefectures, 81 reports/week was converted to 0.31 reports/week per SMI for 259 pediatric medical institutions at fixed locations in Tokyo.

### Application of the Approach to All 47 Prefectures in Japan

As in the case of Tokyo, the model identified the RSV epidemic cycles as periodic oscillations in other prefectures as well, despite the 11 climate zones of Japan (Table 1, Supplemental Figures). The minimum proportion of RSV reports falling below the onset line of RSV season and within the bounds of the sine function was 83.1% (mean 91.3%, median 91.4%, and max 97.1%). The mean number of RSV reports/w at onset of the RSV season for all prefectures was 29.7 reports/week (median 21.0, min 6.0, and max 121.0 reports/week), and the mean number of weekly reports/SMI to define the onset of the RSV season was 0.52 (median 0.53, min 0.22, and max 1.0, Table 1). In general, the onset of the epidemic RSV season in 2017 was more than 1 month earlier than in the previous year, as demonstrated by the data.

## Discussion

Japan is under 11 significantly different climate zones due to its geographical location between the Sea of Japan and the Pacific Ocean, and shape, which spans from north to south. There are 47 prefectures in Japan; the epidemic of RSV infection begins from the south/western area and then tends to spread to the eastern area^1^. The epidemic curve based on RSV reports reaches a peak during summer in Okinawa, the southernmost prefecture and a subtropical region, unlike other prefectures in Japan (12). For this reason, it may be difficult to define the integrated conditions of the onset of the RSV season on a national basis in Japan. Therefore, the conditions of the onset of the RSV season were investigated separately for each prefecture in this study.

First, periodicities of the RSV season in each year were explored for each prefecture using a sinusoidal curve. The fluctuation of the recent epidemic period was found to be periodic every year in all prefectures (Table 1). We then explored the onset of the RSV season, assuming it would come after the trough of the sinusoidal curve for each prefecture.

Previous studies have shown similar modeling approaches for the epidemics of RSV (13–16), in which non-linear regression analyses were applied. In the present study, in addition to the non-linear regression analyses, the model was developed taking account of the following factors and their balance for the epidemic period: (1) the number of RSV reports per week within epidemic periods (a high rate of RSV-reports/w), and (2) the majority of the reports included within the RSV season (a high capture rate).

Generally, if the epidemic period is narrowed down to the vicinity of the peak, although the number of RSV-reports/w will be high, the capture rate will be low, because many RSV infections have been reported before it. Thus, it may be too late to define the epidemic start based on this period definition. Conversely, when the epidemic period is set too early, the capture rate will be sufficiently high, but the number of RSV-reports/w will be too low, and therefore it may be too early to define the onset of the RSV season. Consequently, we considered that a balance between RSV-reports/w and the capture rate of reports is important to detect the onset of the RSV epidemic. To accomplish this task, the RSV-reports/w and capture rate in the epidemic period were quantified to the same scale from 0.0 to 1.0, so that the sum of both could be used as an index. The timing of the maximum value was taken as the epidemic start.

It is worth mentioning that it is mandatory for about 10% of medical institutions (SMIs) to report the RSV-positive cases, and the data are disseminated by the IDWR in Japan, indicating that the weekly reports well represent the real epidemic RSV status in Japan, whereas other case data (hospitalization and RSV-positive rates) used in the previous studies for the modeling in the US were obtained in certain geographical regions (13, 15, 16). Such difference in the data coverage used for the modeling development, as well as that in the modeling methods, make a direct comparison difficult between our study and previous studies.

Criteria for the onset of RSV season for each prefecture were set in the present study, and the results showed a remarkable change in the onset of RSV season by years. The model detected that the onset was 1 or 2 months earlier in 2017 than in 2016. The 2017 RSV epidemic began in July in 34 (72%) prefectures and in June in 4 (8.5%) prefectures (Table 1). The National Institute of Infectious Disease and Tokyo Metropolitan Government announced that the RSV epidemic in 2017 started in July, indicating that our results support the findings of the NIID and the Tokyo Metropolitan Government.

The model also revealed that the changes were small in the southern prefectures. Furthermore, Miyazaki and Kagoshima prefectures, which are located between the temperate zone and the subtropical zone in the south, showed two peaks where RSV prevails, in winter and summer. In Okinawa prefecture, which is located in the subtropical zone at the south end, the epidemic period was in summer. Because of the prefecture-specific tendency in the patterns of RSV cycles, the application of our model for each prefecture will provide more precise and timely information regarding the pattern of RSV seasons.

A limitation of the study is that the model does not predict the onset of the RSV season in advance, and therefore it may not provide timely information regarding its start. However, the model enables us to detect the onset at an early stage so that we can prepare, e.g., to administer palivizumab to infants to prevent the severe RSV infection. Another limitation is that the model may not identify potential onset timing if the number of reported cases fluctuates substantially or if high numbers of cases are observed throughout a year.

The result would be used to prepare for the RSV season by estimating the onset of the next RSV season from the past onsets.

Converting to the reported number per SMI, the differences in the pediatric population by prefecture are corrected, so it would be able to refer to the situation in the neighboring prefecture. For example, many people in the Ibaraki prefecture are commuting to Tokyo.

The result would contribute timely detection of the onset of RSV season even if the season starts earlier than the presumption. It would be expected to help reduce the severe RSV infection in infants by signaling the need for the prompt start of treatment with palivizumab and hence contribute to pediatric and neonatal medical practice.

## Conclusion

In this study, our model detected the onset week of RSV season in 47 prefectures based on the national surveillance data in Japan for 6 years (2012–2017). The model also verified that the onset of RSV season was in early July in 2017. A timely detection of the onset of RSV season is expected to help reduce severe RSV infection in infants by signaling the need for the prompt start of treatment with palivizumab, and thereby contribute to pediatric and neonatal medical practice.

## Data Availability

All datasets generated for this study are included in the manuscript and/or the supplementary files.

## Author Contributions

All authors prepared the manuscripts and approved the manuscript for publication. HY and TH designed the study. TH conducted data analysis. HK, IK, and SK provided efficient advice, from the data analysis planning to obtainment of results.

### Conflict of Interest Statement

AbbVie GK planned the study design and conducted the study. HK, IK, and SK are advisors to AbbVie GK. HY and TH are employees of AbbVie GK.

## Footnotes

^1^https://www.niid.go.jp/niid/ja/rs-virus-m/rs-virus-idwrc/7509-idwrc-1734.html

^2^https://www.jpeds.or.jp/modules/news/index.php?content_id=345

^3^https://www.niid.go.jp/niid/en/survaillance-data-table-english.html

^4^https://www.niid.go.jp/niid/en/calendar-e.html

## Supplementary Material

Data Sheet 1 Detection of the start of the epidemic period of respiratory syncytial virus (RSV) infection using the Infectious Diseases Weekly Report surveillance data (2012-2017 seasons) of 46 prefectures. Black line, number of RSV report; light blue line, RSV epidemic cycle; dotted red line, onset line of RSV season; vertical red line, onset week; vertical blue line, trough of epidemic cycle.
