# Supplementary figures and images for: Detection of the Onset of the Epidemic Period of Respiratory Syncytial Virus Infection in Japan
Source: Front Public Health. 2019 Mar 7;7:39. doi: 10.3389/fpubh.2019.00039 (PMC6425940; doi:10.3389/fpubh.2019.00039)

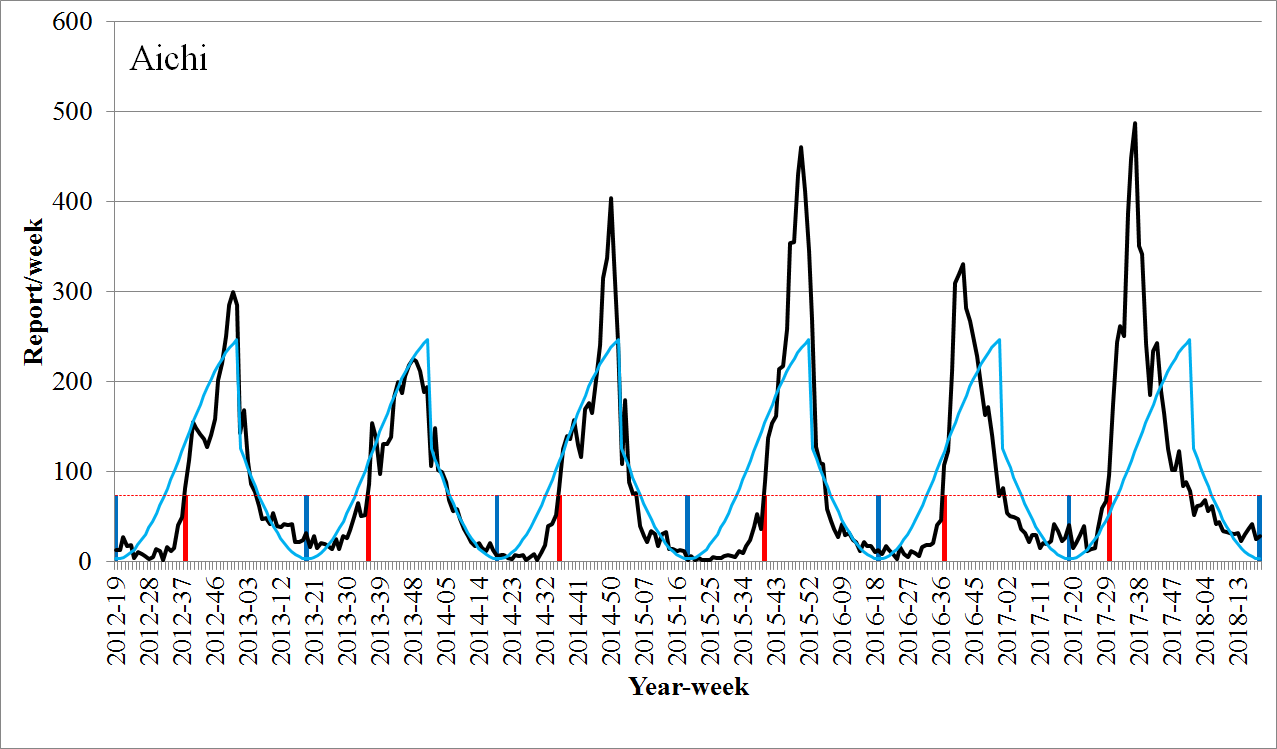

Supplement: Data Sheet 1 — Detection of the start of the epidemic period of respiratory syncytial virus (RSV) infection using the Infectious Diseases Weekly Report surveillance data (2012-2017 seasons) of 46 prefectures. Black line, number of RSV report; light blue line, RSV epidemic cycle; dotted red line, onset line of RSV season; vertical red line, onset week; vertical blue line, trough of epidemic cycle. [file Data_Sheet_1.ZIP › Yamagami et al_Supplementary Figures/Aichi.png]

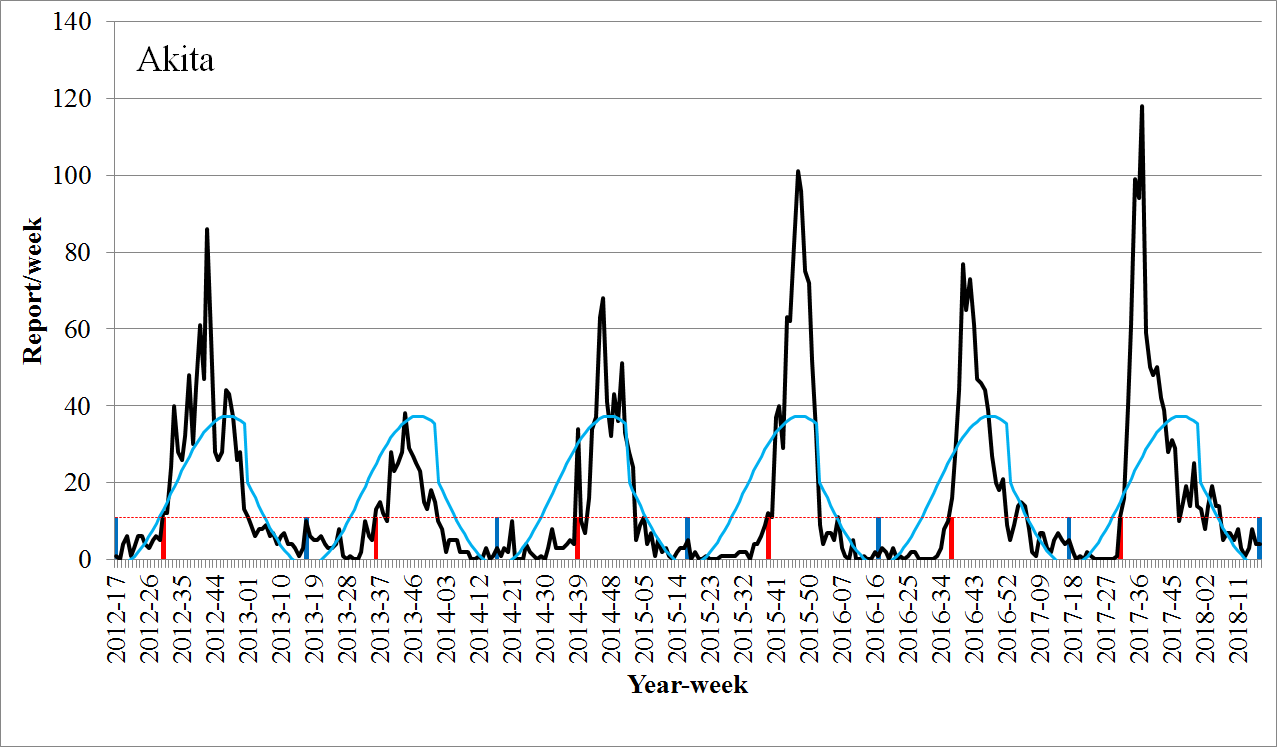

Supplement: Data Sheet 1 — Detection of the start of the epidemic period of respiratory syncytial virus (RSV) infection using the Infectious Diseases Weekly Report surveillance data (2012-2017 seasons) of 46 prefectures. Black line, number of RSV report; light blue line, RSV epidemic cycle; dotted red line, onset line of RSV season; vertical red line, onset week; vertical blue line, trough of epidemic cycle. [file Data_Sheet_1.ZIP › Yamagami et al_Supplementary Figures/Akita.png]

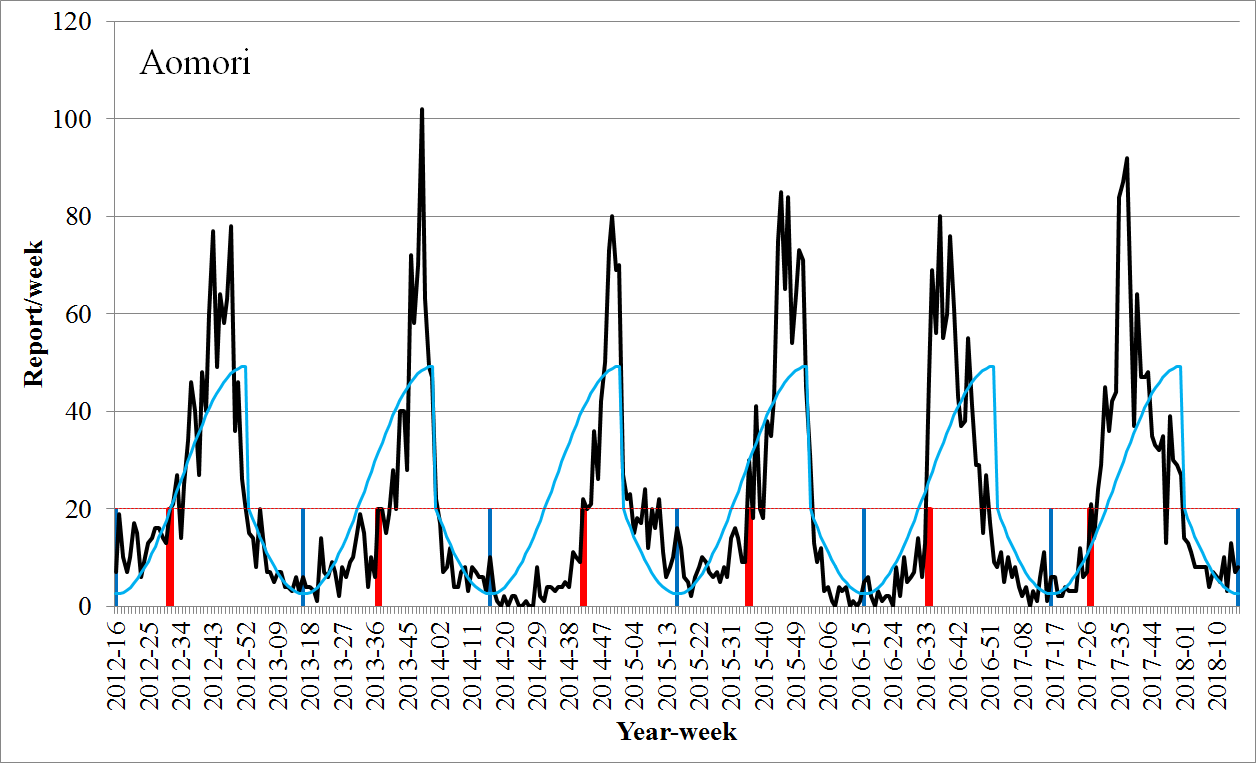

Supplement: Data Sheet 1 — Detection of the start of the epidemic period of respiratory syncytial virus (RSV) infection using the Infectious Diseases Weekly Report surveillance data (2012-2017 seasons) of 46 prefectures. Black line, number of RSV report; light blue line, RSV epidemic cycle; dotted red line, onset line of RSV season; vertical red line, onset week; vertical blue line, trough of epidemic cycle. [file Data_Sheet_1.ZIP › Yamagami et al_Supplementary Figures/Aomori.png]

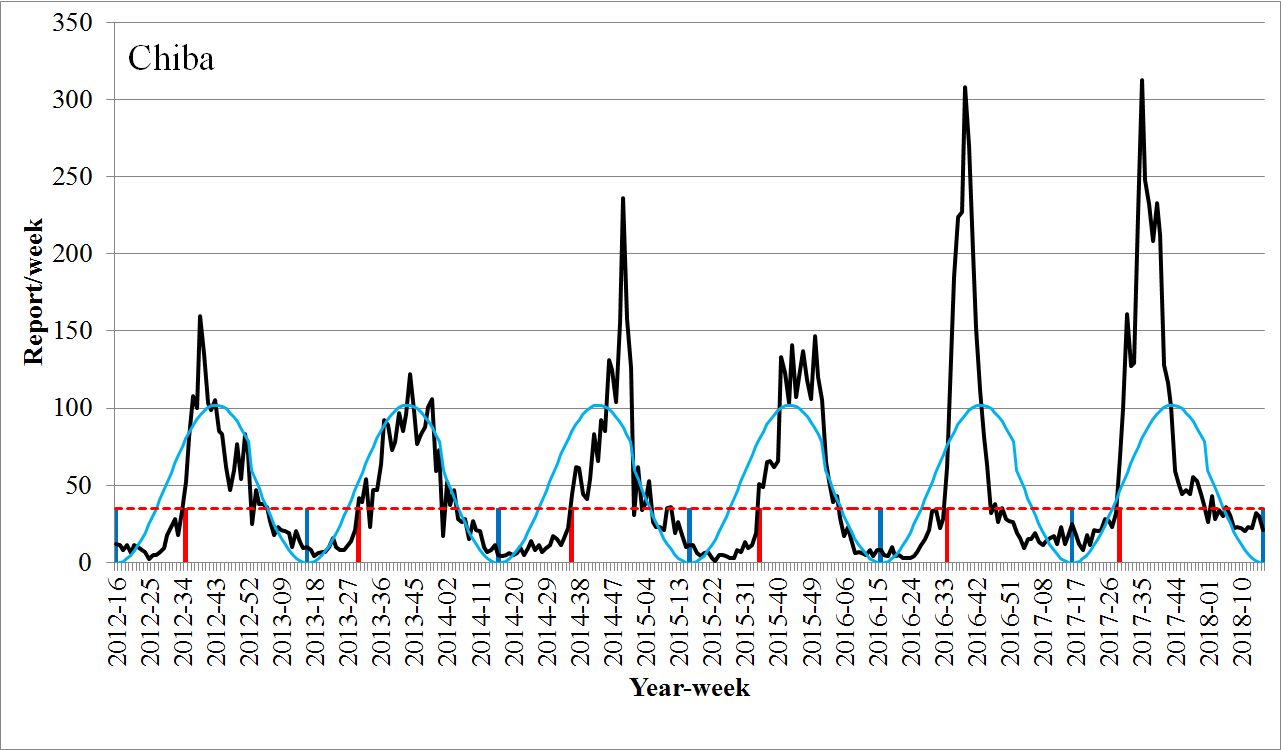

Supplement: Data Sheet 1 — Detection of the start of the epidemic period of respiratory syncytial virus (RSV) infection using the Infectious Diseases Weekly Report surveillance data (2012-2017 seasons) of 46 prefectures. Black line, number of RSV report; light blue line, RSV epidemic cycle; dotted red line, onset line of RSV season; vertical red line, onset week; vertical blue line, trough of epidemic cycle. [file Data_Sheet_1.ZIP › Yamagami et al_Supplementary Figures/Chiba.png]

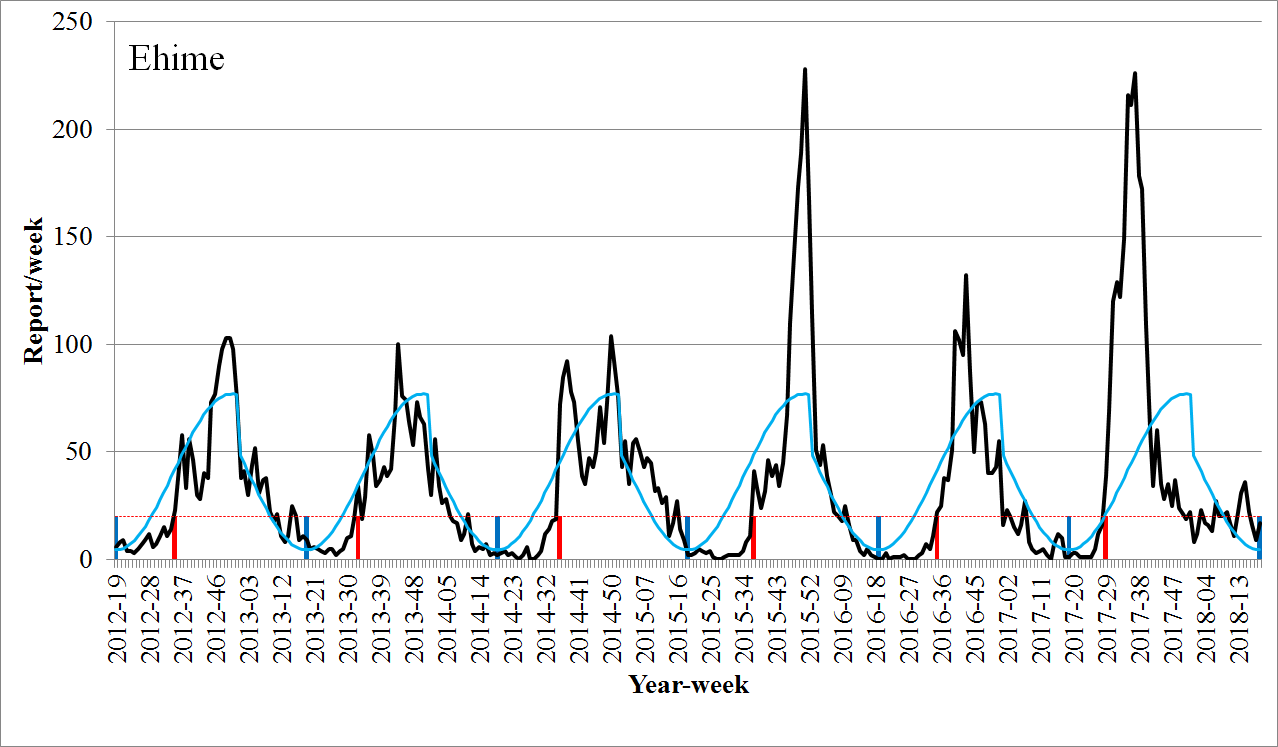

Supplement: Data Sheet 1 — Detection of the start of the epidemic period of respiratory syncytial virus (RSV) infection using the Infectious Diseases Weekly Report surveillance data (2012-2017 seasons) of 46 prefectures. Black line, number of RSV report; light blue line, RSV epidemic cycle; dotted red line, onset line of RSV season; vertical red line, onset week; vertical blue line, trough of epidemic cycle. [file Data_Sheet_1.ZIP › Yamagami et al_Supplementary Figures/Ehime.png]

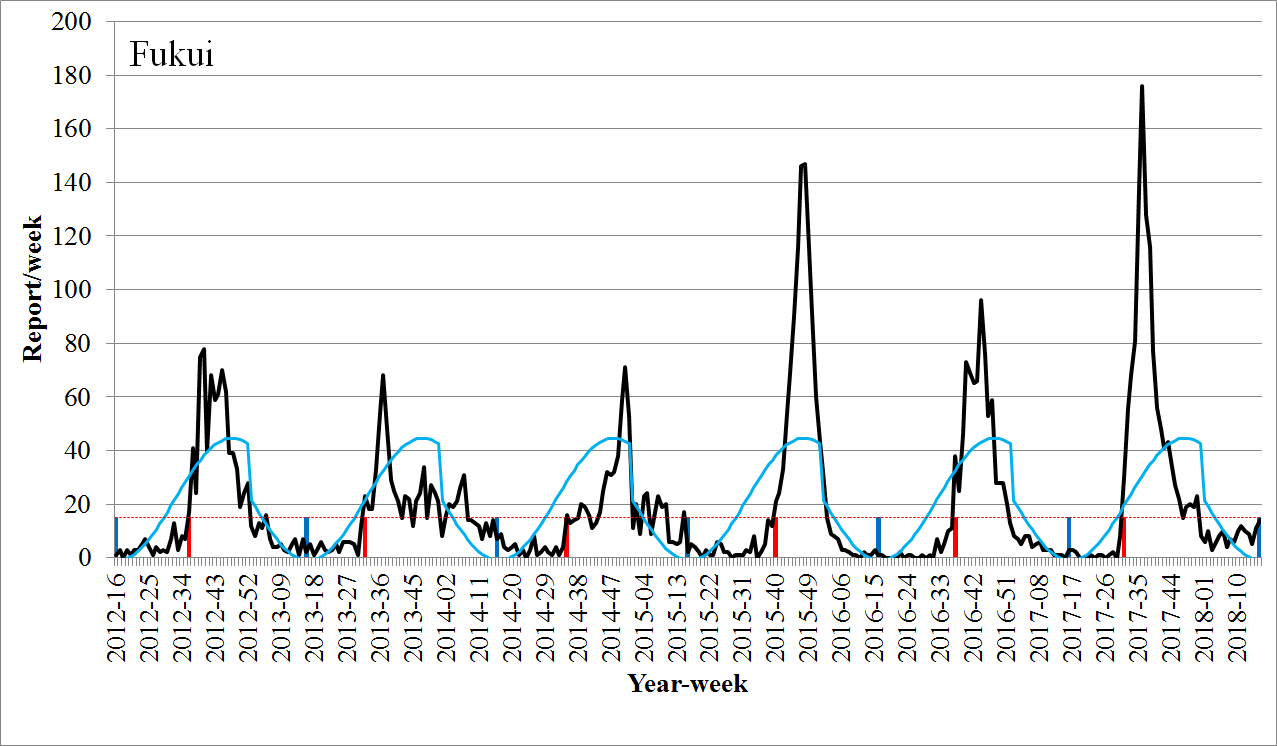

Supplement: Data Sheet 1 — Detection of the start of the epidemic period of respiratory syncytial virus (RSV) infection using the Infectious Diseases Weekly Report surveillance data (2012-2017 seasons) of 46 prefectures. Black line, number of RSV report; light blue line, RSV epidemic cycle; dotted red line, onset line of RSV season; vertical red line, onset week; vertical blue line, trough of epidemic cycle. [file Data_Sheet_1.ZIP › Yamagami et al_Supplementary Figures/Fukui.png]

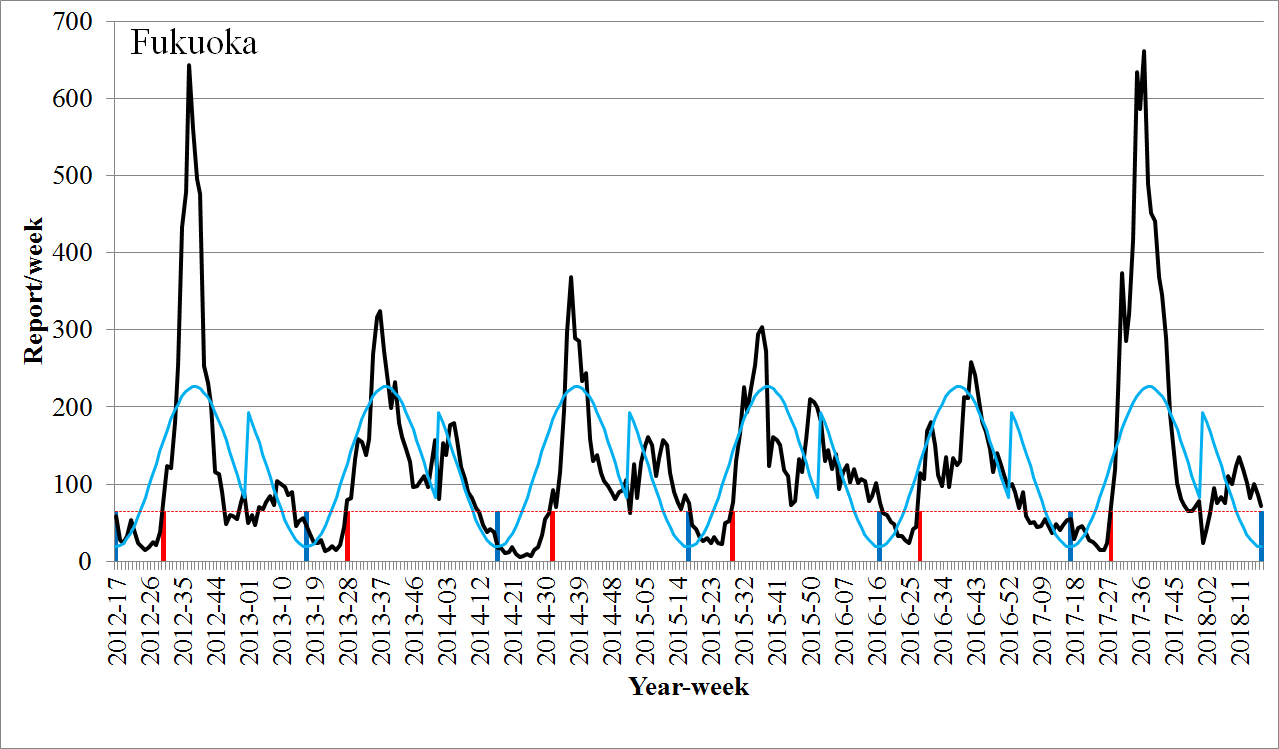

Supplement: Data Sheet 1 — Detection of the start of the epidemic period of respiratory syncytial virus (RSV) infection using the Infectious Diseases Weekly Report surveillance data (2012-2017 seasons) of 46 prefectures. Black line, number of RSV report; light blue line, RSV epidemic cycle; dotted red line, onset line of RSV season; vertical red line, onset week; vertical blue line, trough of epidemic cycle. [file Data_Sheet_1.ZIP › Yamagami et al_Supplementary Figures/Fukuoka.png]

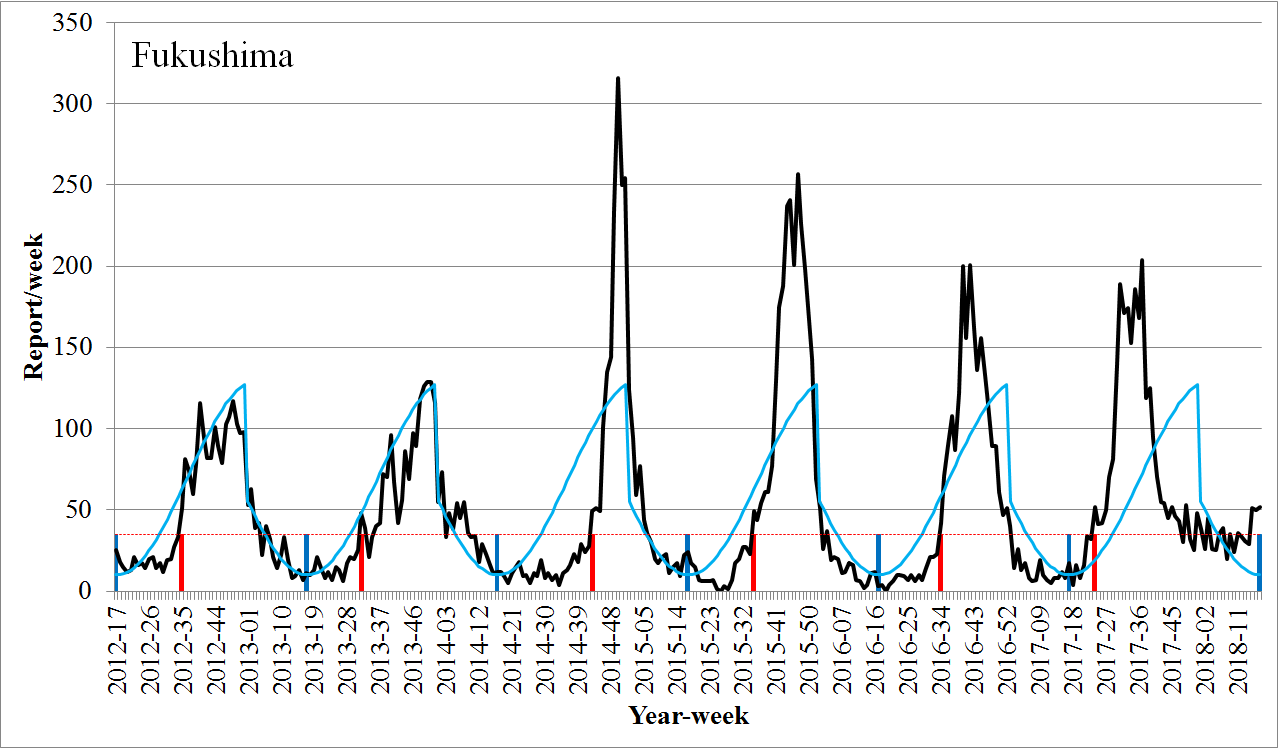

Supplement: Data Sheet 1 — Detection of the start of the epidemic period of respiratory syncytial virus (RSV) infection using the Infectious Diseases Weekly Report surveillance data (2012-2017 seasons) of 46 prefectures. Black line, number of RSV report; light blue line, RSV epidemic cycle; dotted red line, onset line of RSV season; vertical red line, onset week; vertical blue line, trough of epidemic cycle. [file Data_Sheet_1.ZIP › Yamagami et al_Supplementary Figures/Fukushima.png]

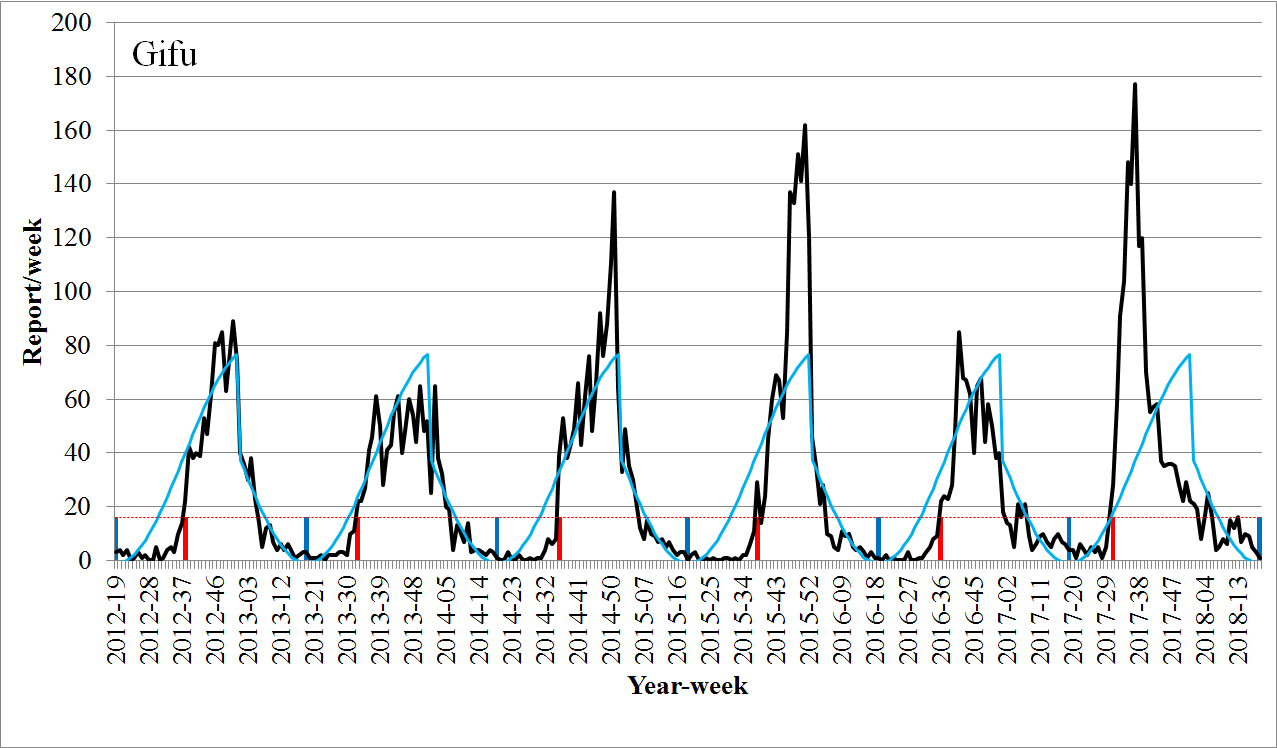

Supplement: Data Sheet 1 — Detection of the start of the epidemic period of respiratory syncytial virus (RSV) infection using the Infectious Diseases Weekly Report surveillance data (2012-2017 seasons) of 46 prefectures. Black line, number of RSV report; light blue line, RSV epidemic cycle; dotted red line, onset line of RSV season; vertical red line, onset week; vertical blue line, trough of epidemic cycle. [file Data_Sheet_1.ZIP › Yamagami et al_Supplementary Figures/Gifu.png]

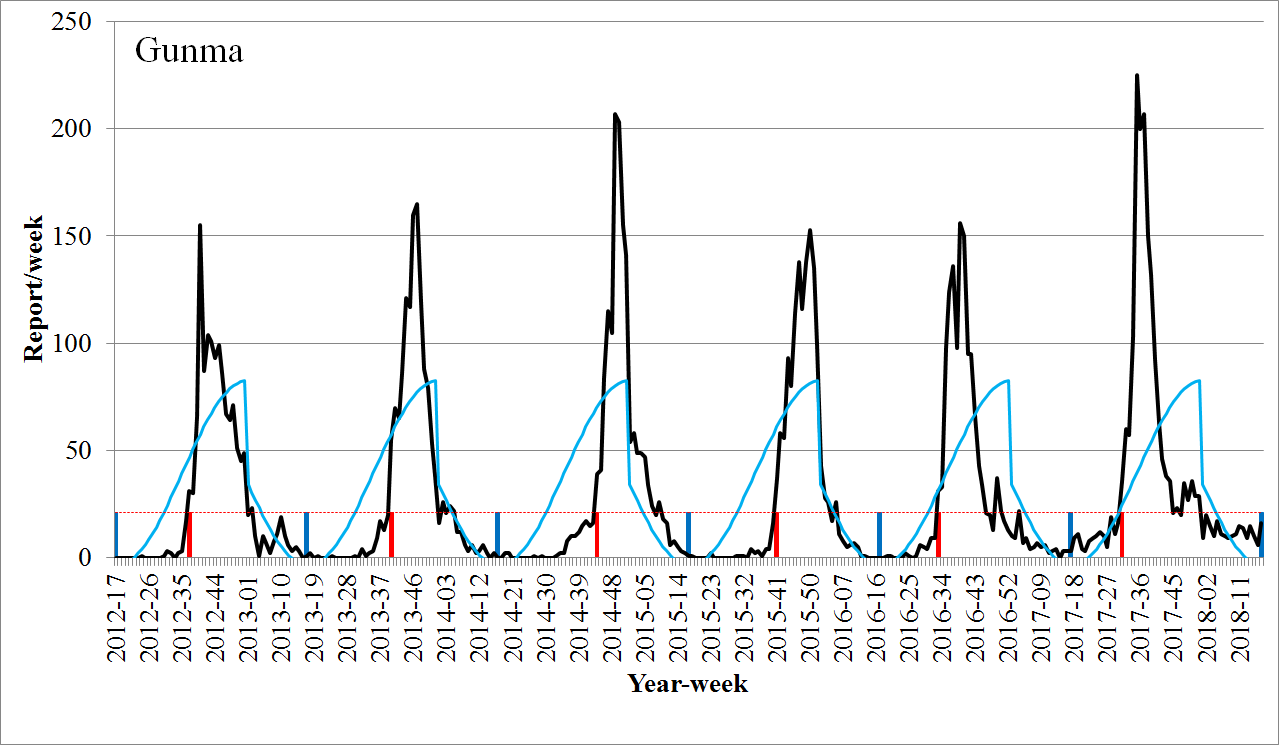

Supplement: Data Sheet 1 — Detection of the start of the epidemic period of respiratory syncytial virus (RSV) infection using the Infectious Diseases Weekly Report surveillance data (2012-2017 seasons) of 46 prefectures. Black line, number of RSV report; light blue line, RSV epidemic cycle; dotted red line, onset line of RSV season; vertical red line, onset week; vertical blue line, trough of epidemic cycle. [file Data_Sheet_1.ZIP › Yamagami et al_Supplementary Figures/Gunma.png]

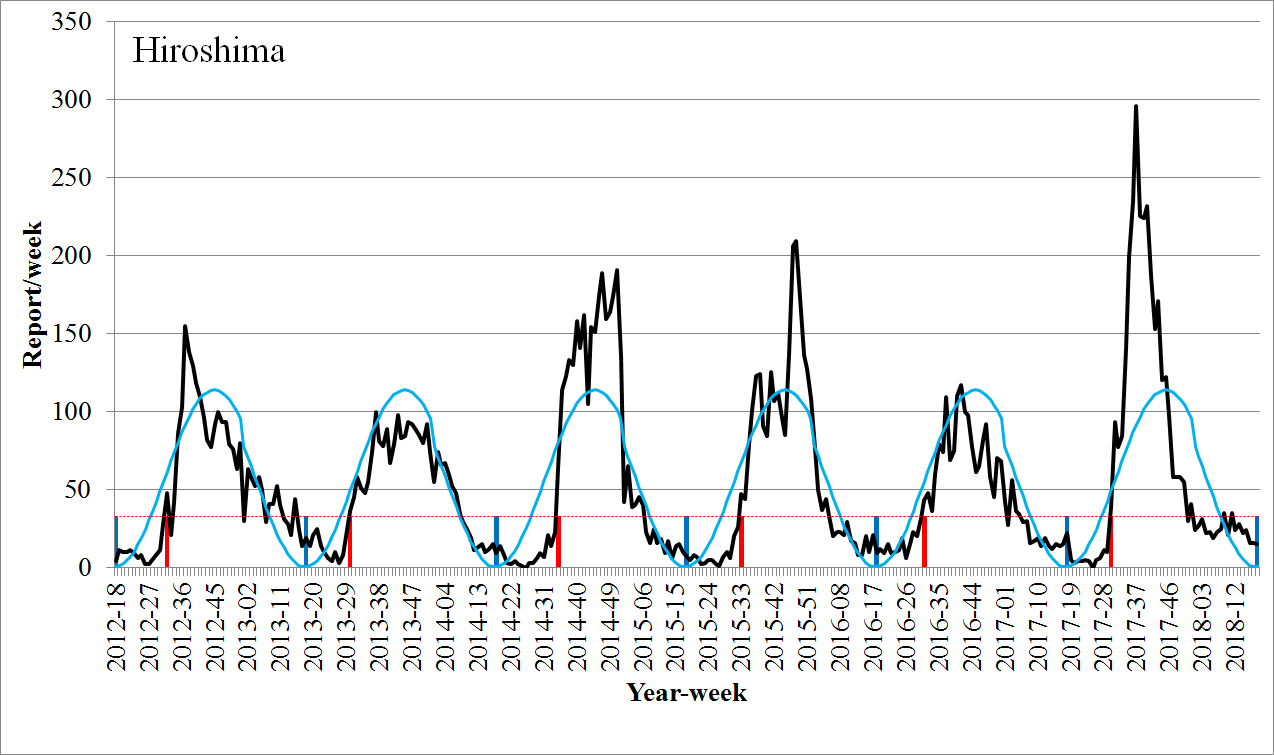

Supplement: Data Sheet 1 — Detection of the start of the epidemic period of respiratory syncytial virus (RSV) infection using the Infectious Diseases Weekly Report surveillance data (2012-2017 seasons) of 46 prefectures. Black line, number of RSV report; light blue line, RSV epidemic cycle; dotted red line, onset line of RSV season; vertical red line, onset week; vertical blue line, trough of epidemic cycle. [file Data_Sheet_1.ZIP › Yamagami et al_Supplementary Figures/Hiroshima.png]

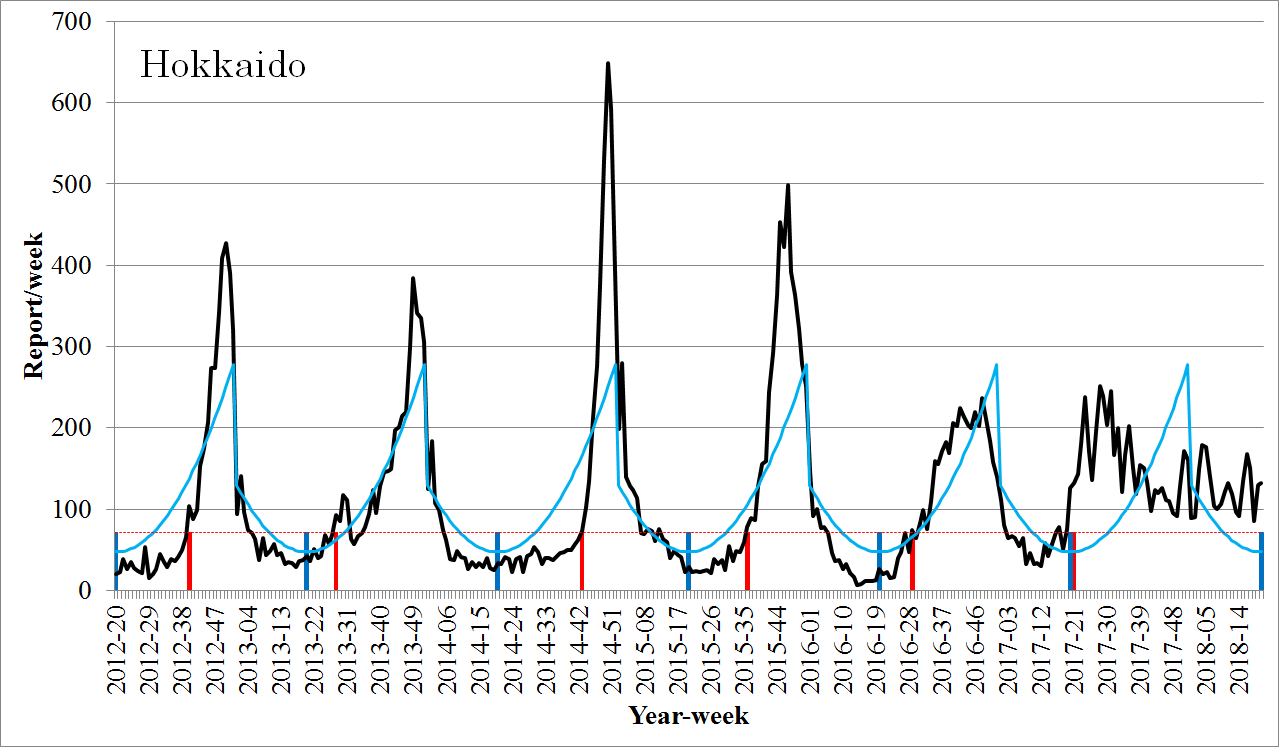

Supplement: Data Sheet 1 — Detection of the start of the epidemic period of respiratory syncytial virus (RSV) infection using the Infectious Diseases Weekly Report surveillance data (2012-2017 seasons) of 46 prefectures. Black line, number of RSV report; light blue line, RSV epidemic cycle; dotted red line, onset line of RSV season; vertical red line, onset week; vertical blue line, trough of epidemic cycle. [file Data_Sheet_1.ZIP › Yamagami et al_Supplementary Figures/Hokkaido.png]

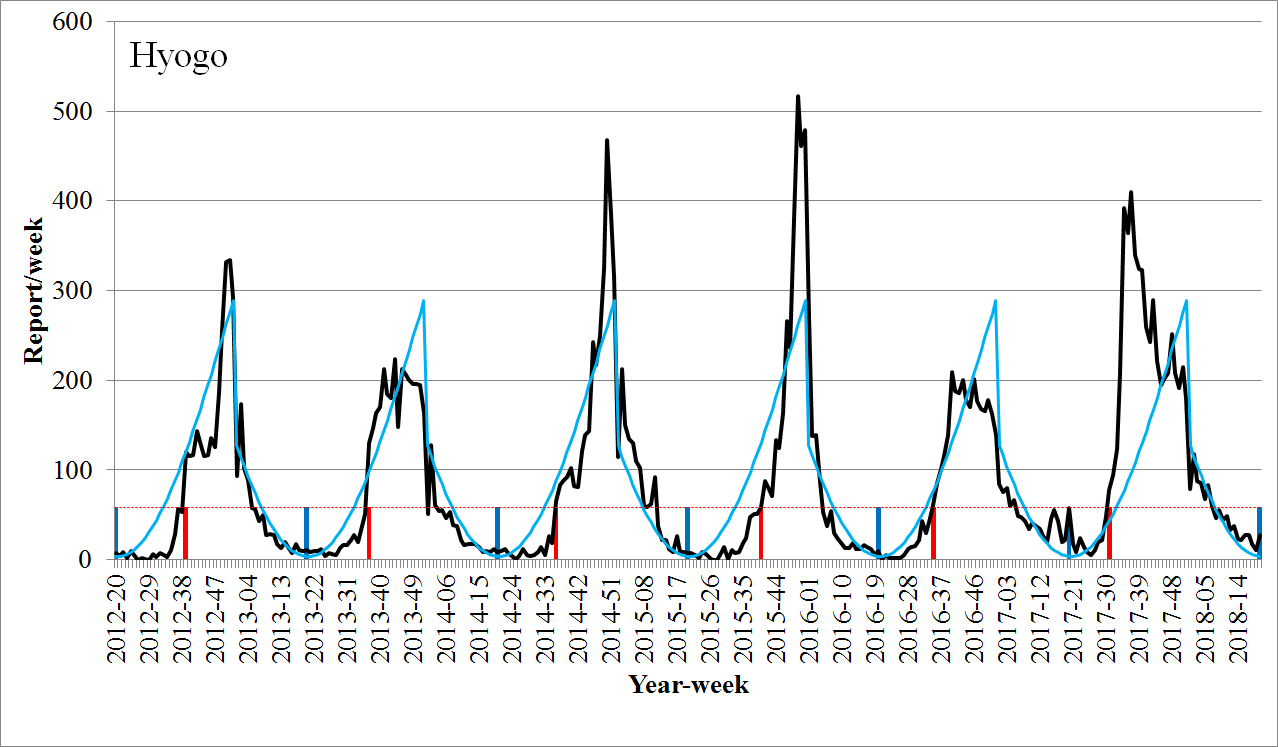

Supplement: Data Sheet 1 — Detection of the start of the epidemic period of respiratory syncytial virus (RSV) infection using the Infectious Diseases Weekly Report surveillance data (2012-2017 seasons) of 46 prefectures. Black line, number of RSV report; light blue line, RSV epidemic cycle; dotted red line, onset line of RSV season; vertical red line, onset week; vertical blue line, trough of epidemic cycle. [file Data_Sheet_1.ZIP › Yamagami et al_Supplementary Figures/Hyogo.png]

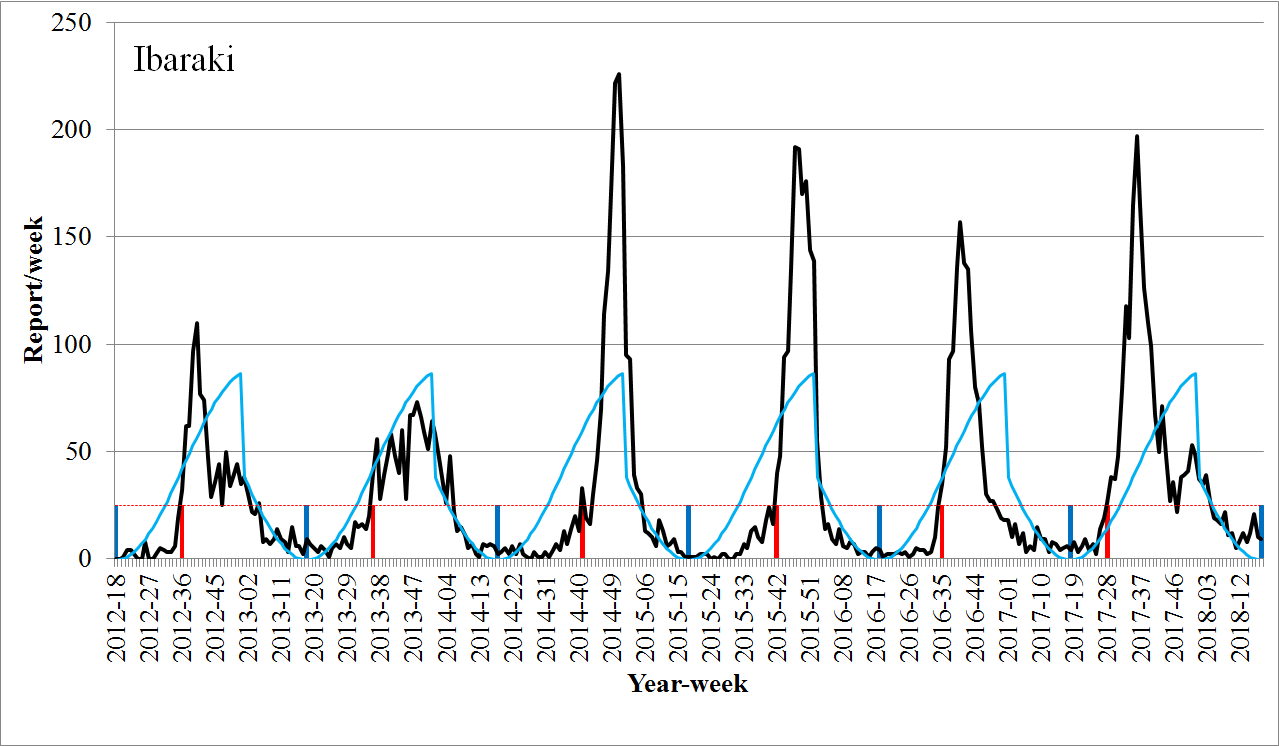

Supplement: Data Sheet 1 — Detection of the start of the epidemic period of respiratory syncytial virus (RSV) infection using the Infectious Diseases Weekly Report surveillance data (2012-2017 seasons) of 46 prefectures. Black line, number of RSV report; light blue line, RSV epidemic cycle; dotted red line, onset line of RSV season; vertical red line, onset week; vertical blue line, trough of epidemic cycle. [file Data_Sheet_1.ZIP › Yamagami et al_Supplementary Figures/Ibaraki.png]

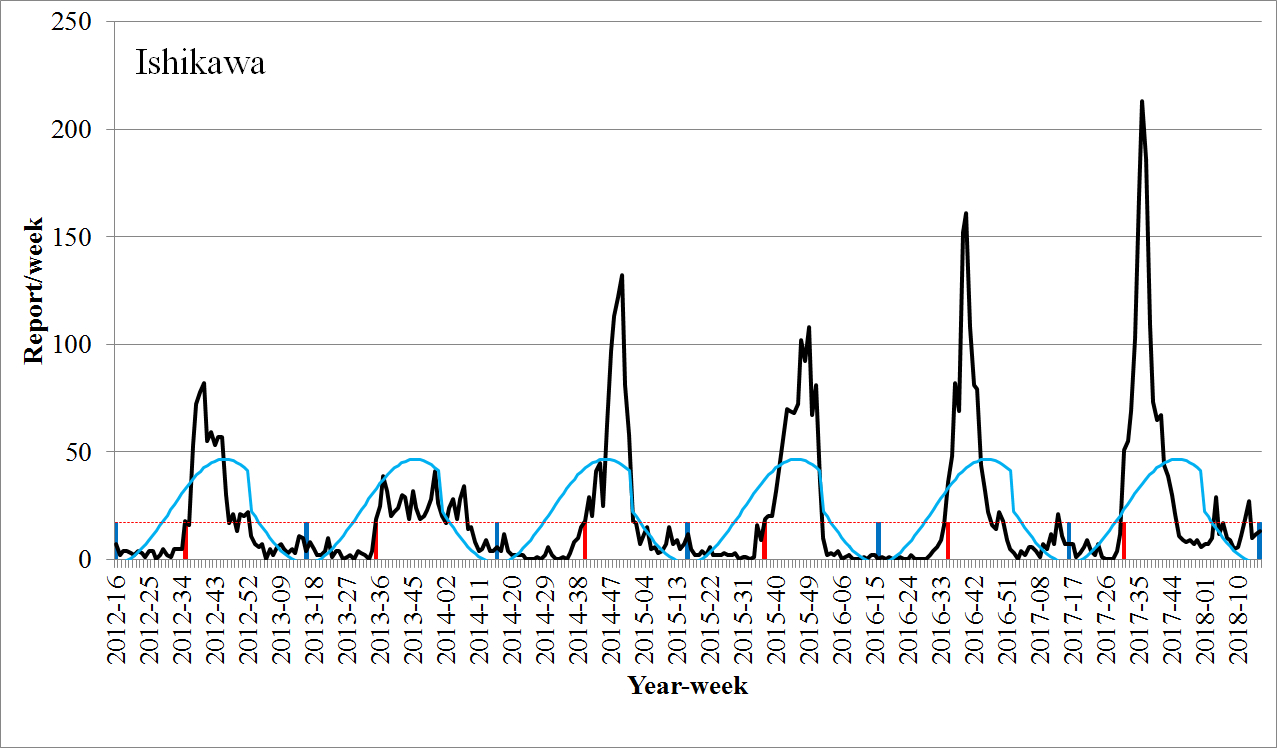

Supplement: Data Sheet 1 — Detection of the start of the epidemic period of respiratory syncytial virus (RSV) infection using the Infectious Diseases Weekly Report surveillance data (2012-2017 seasons) of 46 prefectures. Black line, number of RSV report; light blue line, RSV epidemic cycle; dotted red line, onset line of RSV season; vertical red line, onset week; vertical blue line, trough of epidemic cycle. [file Data_Sheet_1.ZIP › Yamagami et al_Supplementary Figures/Ishikawa.png]

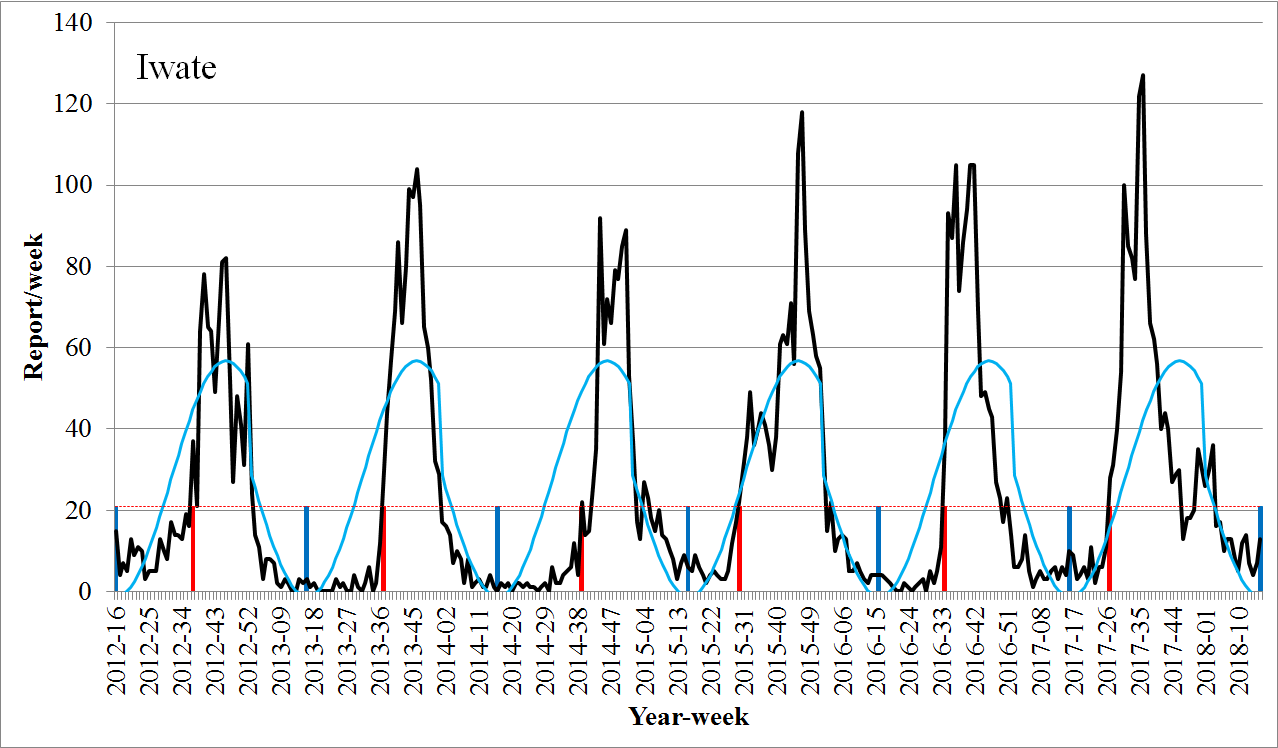

Supplement: Data Sheet 1 — Detection of the start of the epidemic period of respiratory syncytial virus (RSV) infection using the Infectious Diseases Weekly Report surveillance data (2012-2017 seasons) of 46 prefectures. Black line, number of RSV report; light blue line, RSV epidemic cycle; dotted red line, onset line of RSV season; vertical red line, onset week; vertical blue line, trough of epidemic cycle. [file Data_Sheet_1.ZIP › Yamagami et al_Supplementary Figures/Iwate.png]

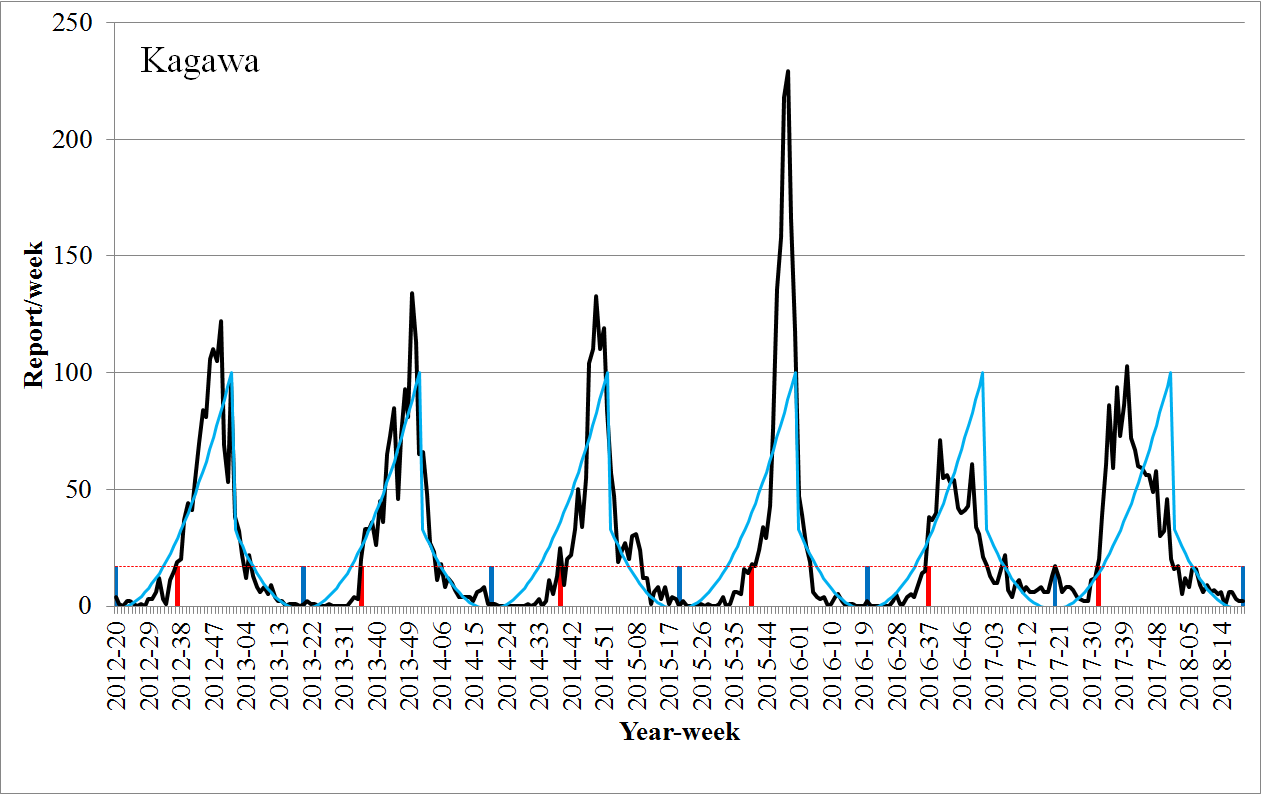

Supplement: Data Sheet 1 — Detection of the start of the epidemic period of respiratory syncytial virus (RSV) infection using the Infectious Diseases Weekly Report surveillance data (2012-2017 seasons) of 46 prefectures. Black line, number of RSV report; light blue line, RSV epidemic cycle; dotted red line, onset line of RSV season; vertical red line, onset week; vertical blue line, trough of epidemic cycle. [file Data_Sheet_1.ZIP › Yamagami et al_Supplementary Figures/Kagawa.png]

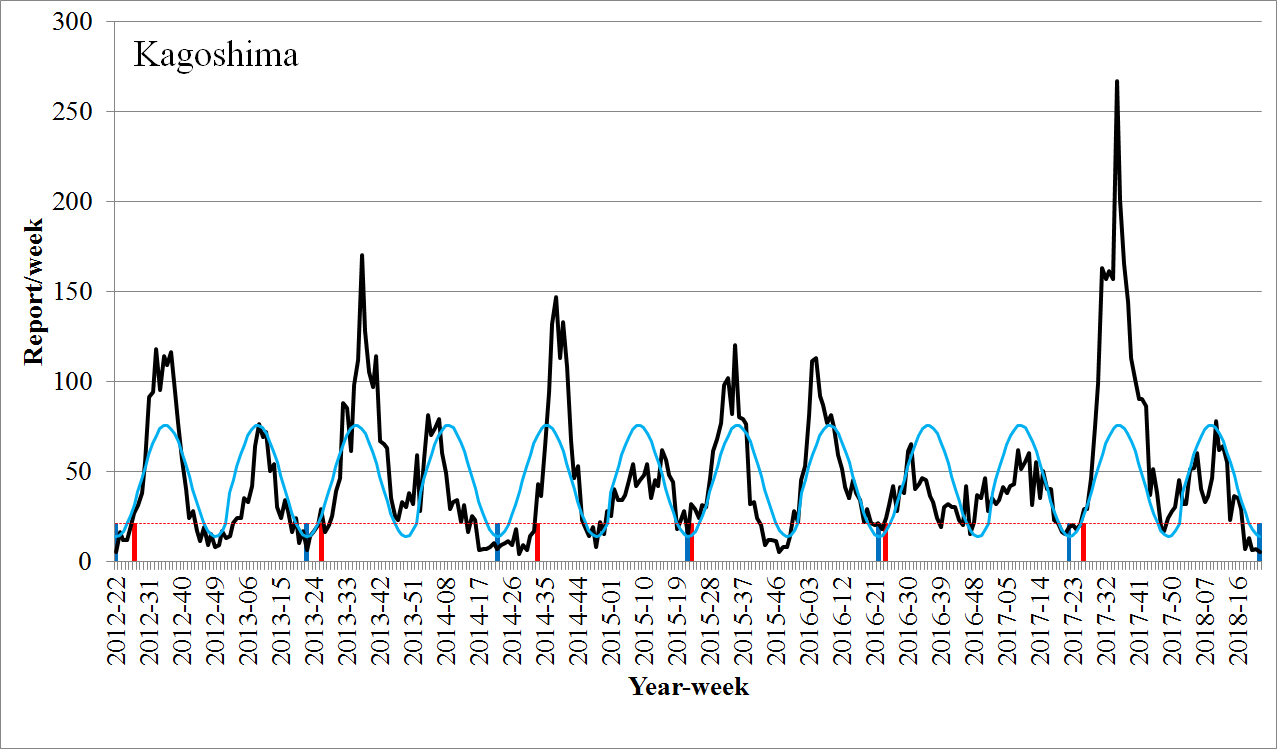

Supplement: Data Sheet 1 — Detection of the start of the epidemic period of respiratory syncytial virus (RSV) infection using the Infectious Diseases Weekly Report surveillance data (2012-2017 seasons) of 46 prefectures. Black line, number of RSV report; light blue line, RSV epidemic cycle; dotted red line, onset line of RSV season; vertical red line, onset week; vertical blue line, trough of epidemic cycle. [file Data_Sheet_1.ZIP › Yamagami et al_Supplementary Figures/Kagoshima.png]

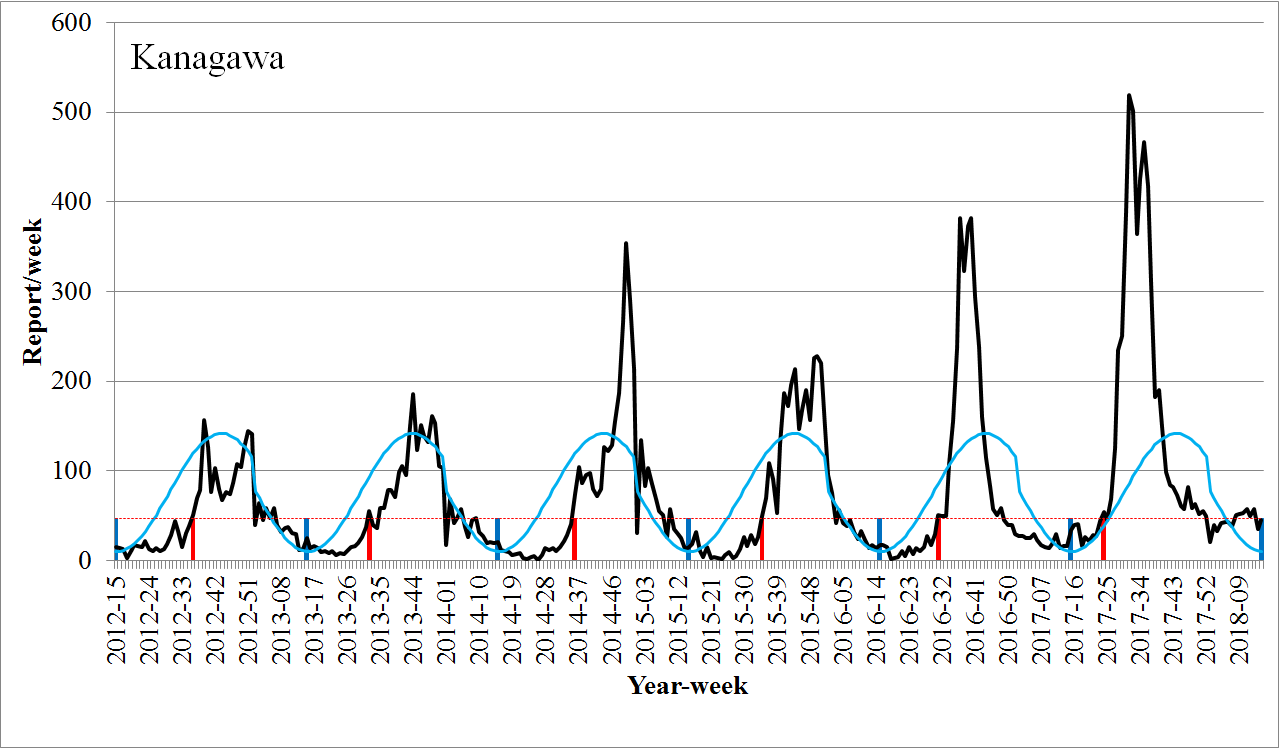

Supplement: Data Sheet 1 — Detection of the start of the epidemic period of respiratory syncytial virus (RSV) infection using the Infectious Diseases Weekly Report surveillance data (2012-2017 seasons) of 46 prefectures. Black line, number of RSV report; light blue line, RSV epidemic cycle; dotted red line, onset line of RSV season; vertical red line, onset week; vertical blue line, trough of epidemic cycle. [file Data_Sheet_1.ZIP › Yamagami et al_Supplementary Figures/Kanagawa.png]

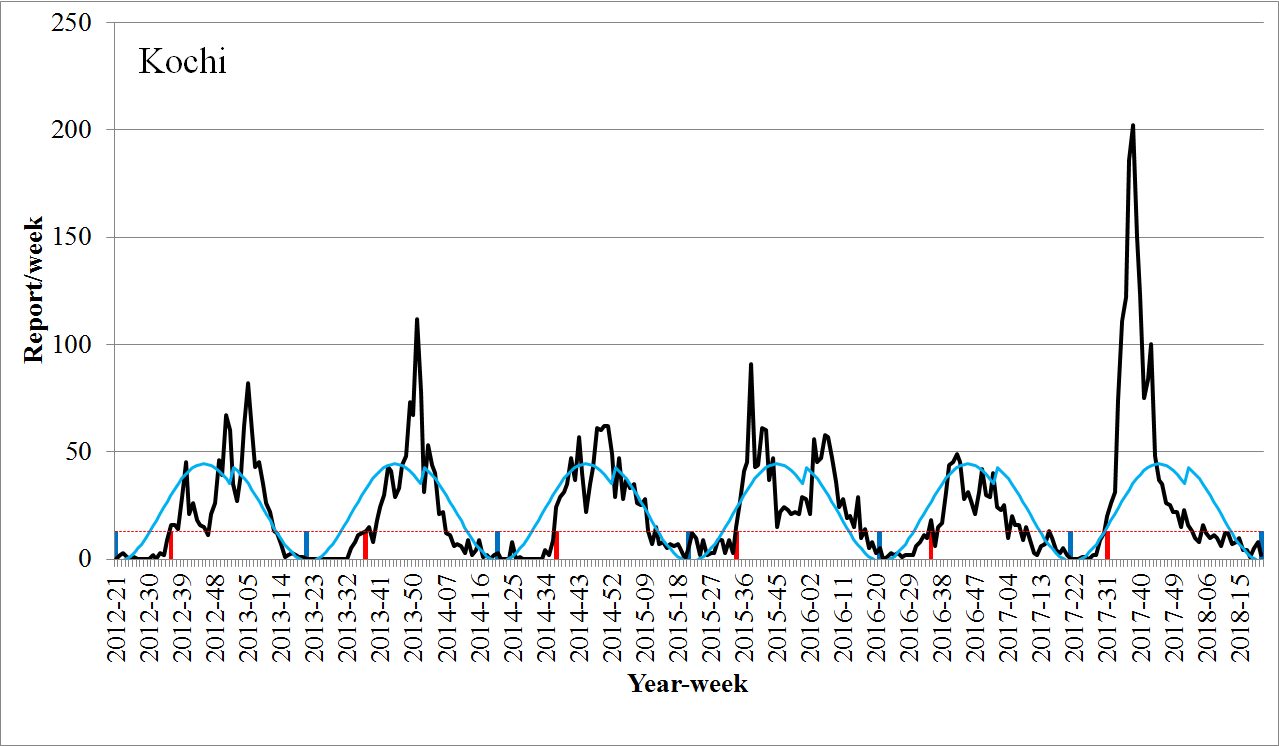

Supplement: Data Sheet 1 — Detection of the start of the epidemic period of respiratory syncytial virus (RSV) infection using the Infectious Diseases Weekly Report surveillance data (2012-2017 seasons) of 46 prefectures. Black line, number of RSV report; light blue line, RSV epidemic cycle; dotted red line, onset line of RSV season; vertical red line, onset week; vertical blue line, trough of epidemic cycle. [file Data_Sheet_1.ZIP › Yamagami et al_Supplementary Figures/Kochi.png]

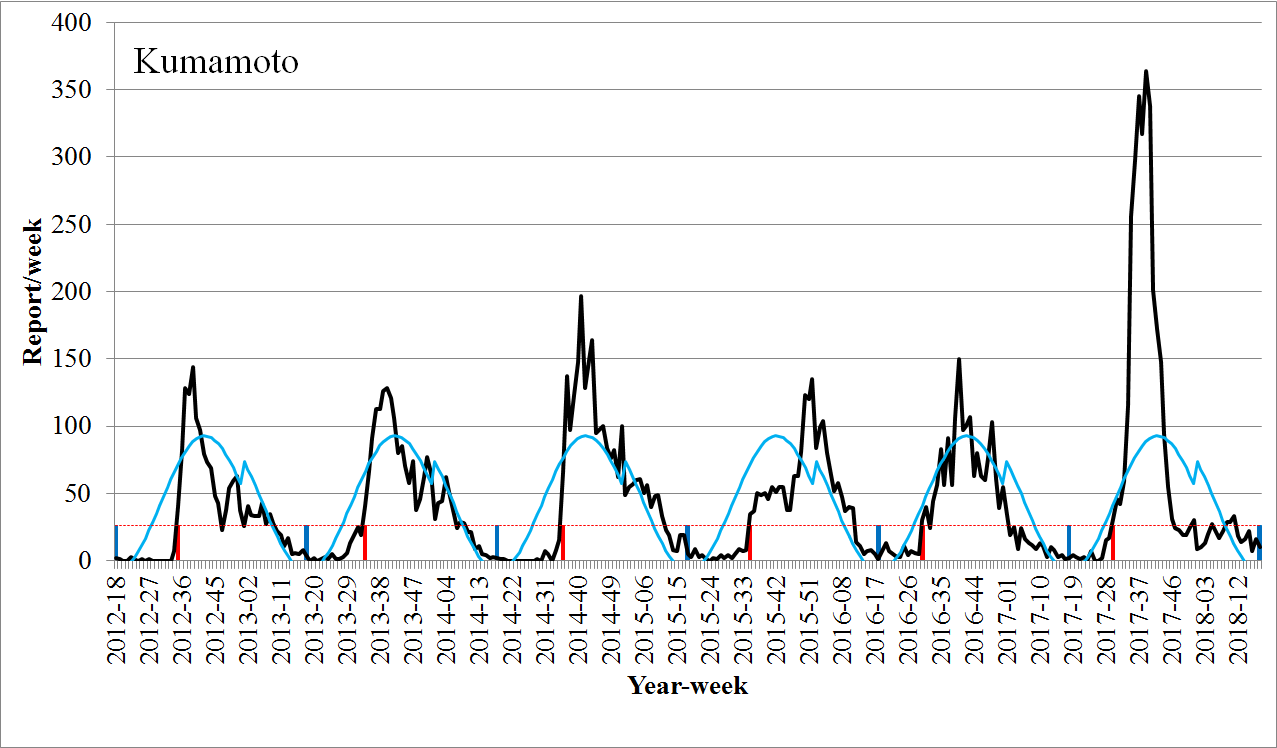

Supplement: Data Sheet 1 — Detection of the start of the epidemic period of respiratory syncytial virus (RSV) infection using the Infectious Diseases Weekly Report surveillance data (2012-2017 seasons) of 46 prefectures. Black line, number of RSV report; light blue line, RSV epidemic cycle; dotted red line, onset line of RSV season; vertical red line, onset week; vertical blue line, trough of epidemic cycle. [file Data_Sheet_1.ZIP › Yamagami et al_Supplementary Figures/Kumamoto.png]

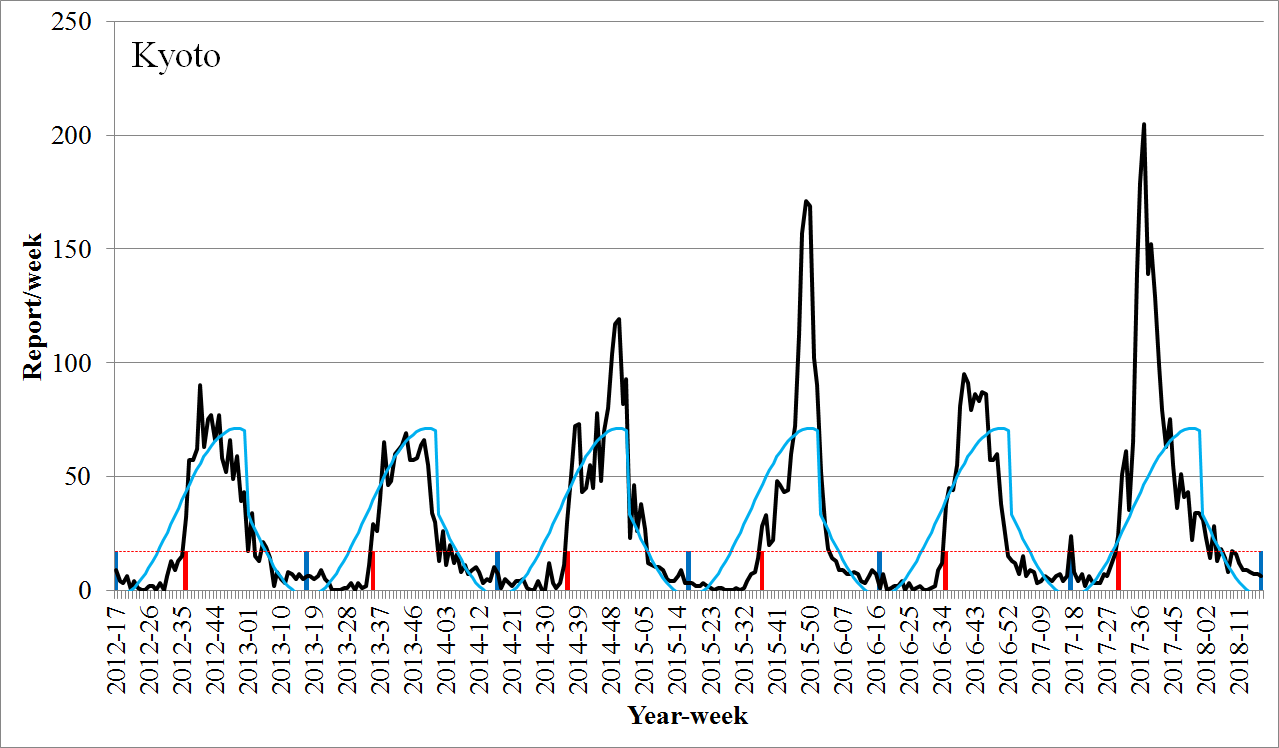

Supplement: Data Sheet 1 — Detection of the start of the epidemic period of respiratory syncytial virus (RSV) infection using the Infectious Diseases Weekly Report surveillance data (2012-2017 seasons) of 46 prefectures. Black line, number of RSV report; light blue line, RSV epidemic cycle; dotted red line, onset line of RSV season; vertical red line, onset week; vertical blue line, trough of epidemic cycle. [file Data_Sheet_1.ZIP › Yamagami et al_Supplementary Figures/Kyoto.png]

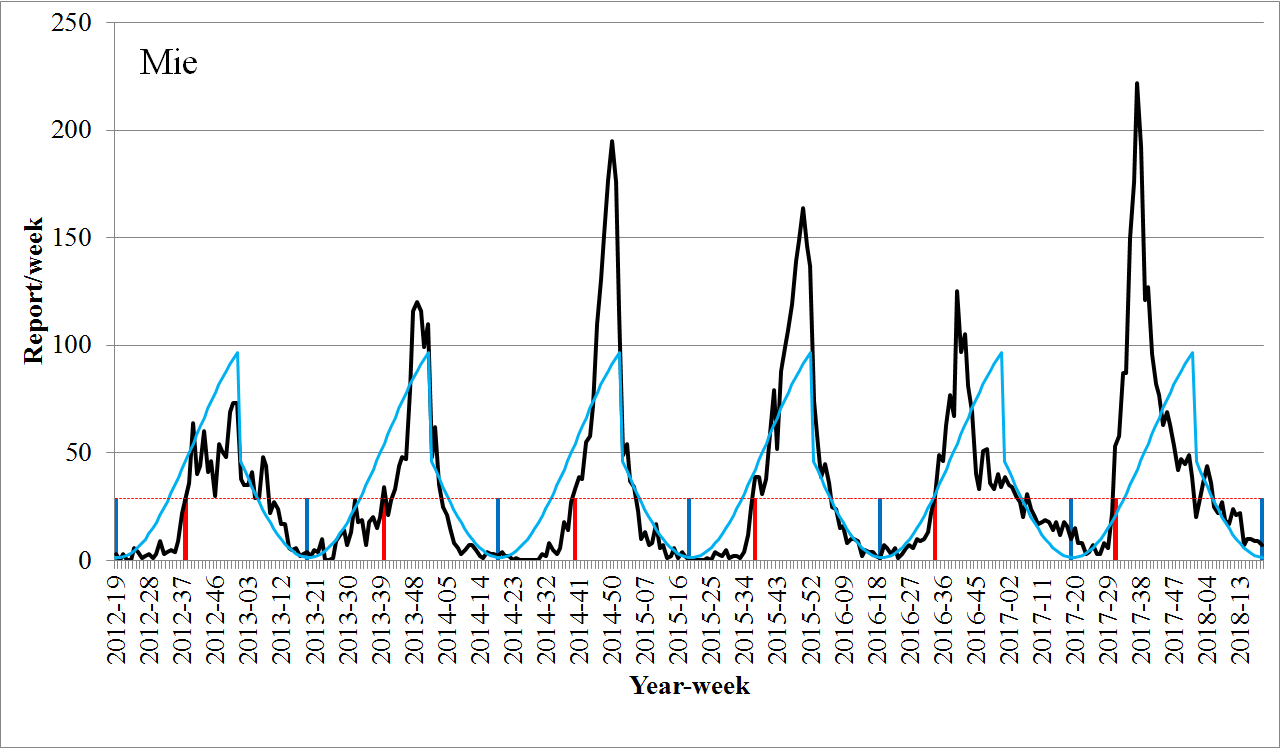

Supplement: Data Sheet 1 — Detection of the start of the epidemic period of respiratory syncytial virus (RSV) infection using the Infectious Diseases Weekly Report surveillance data (2012-2017 seasons) of 46 prefectures. Black line, number of RSV report; light blue line, RSV epidemic cycle; dotted red line, onset line of RSV season; vertical red line, onset week; vertical blue line, trough of epidemic cycle. [file Data_Sheet_1.ZIP › Yamagami et al_Supplementary Figures/Mie.png]

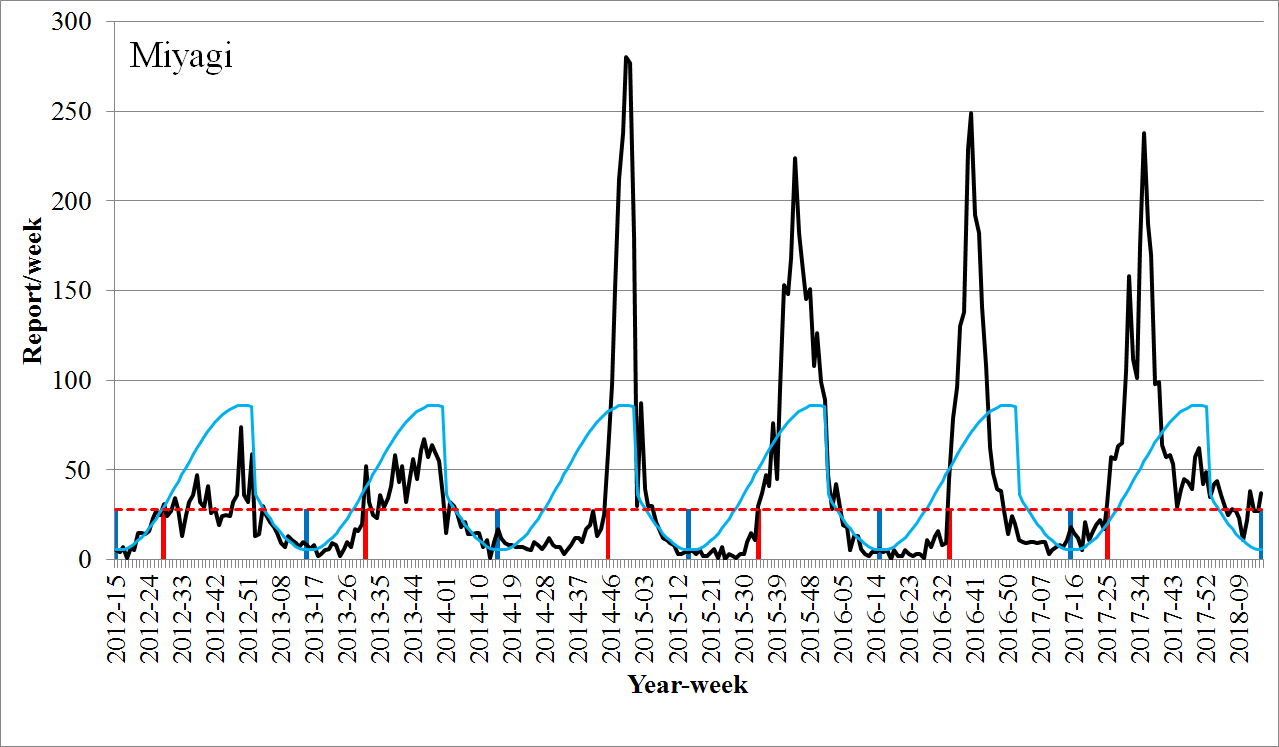

Supplement: Data Sheet 1 — Detection of the start of the epidemic period of respiratory syncytial virus (RSV) infection using the Infectious Diseases Weekly Report surveillance data (2012-2017 seasons) of 46 prefectures. Black line, number of RSV report; light blue line, RSV epidemic cycle; dotted red line, onset line of RSV season; vertical red line, onset week; vertical blue line, trough of epidemic cycle. [file Data_Sheet_1.ZIP › Yamagami et al_Supplementary Figures/Miyagi.png]

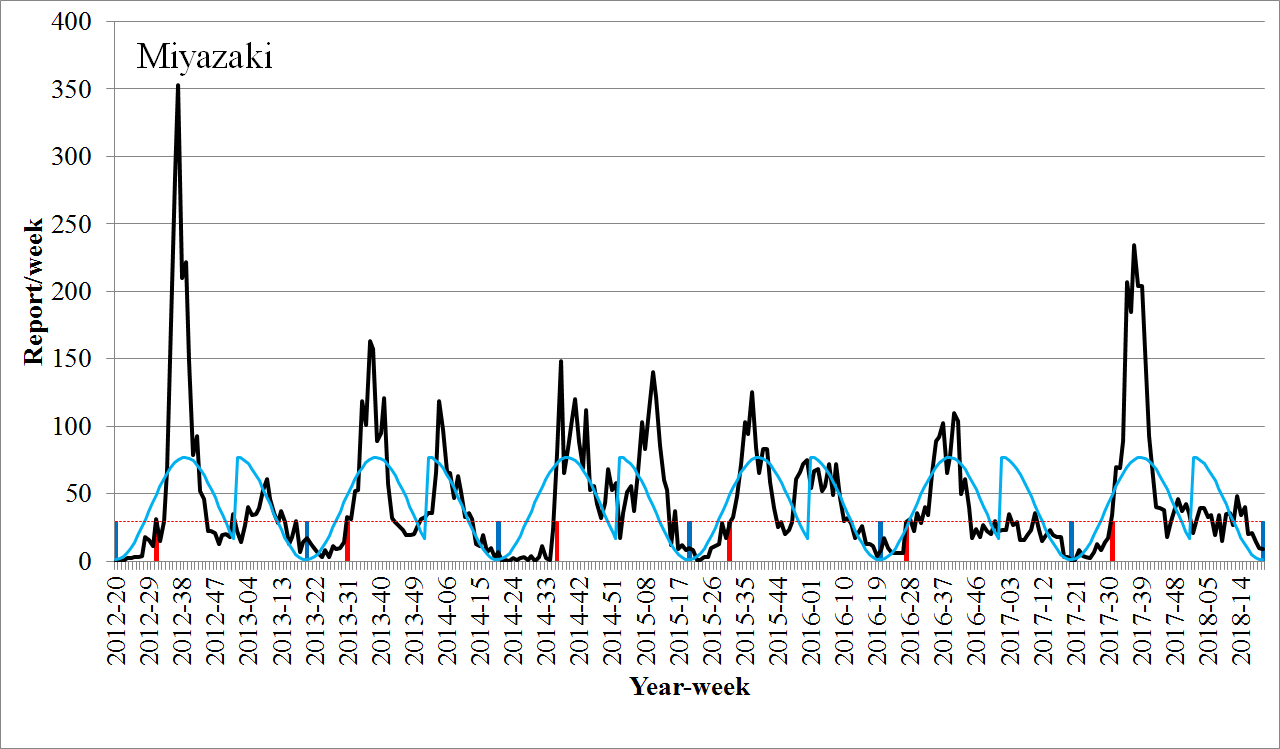

Supplement: Data Sheet 1 — Detection of the start of the epidemic period of respiratory syncytial virus (RSV) infection using the Infectious Diseases Weekly Report surveillance data (2012-2017 seasons) of 46 prefectures. Black line, number of RSV report; light blue line, RSV epidemic cycle; dotted red line, onset line of RSV season; vertical red line, onset week; vertical blue line, trough of epidemic cycle. [file Data_Sheet_1.ZIP › Yamagami et al_Supplementary Figures/Miyazaki.png]

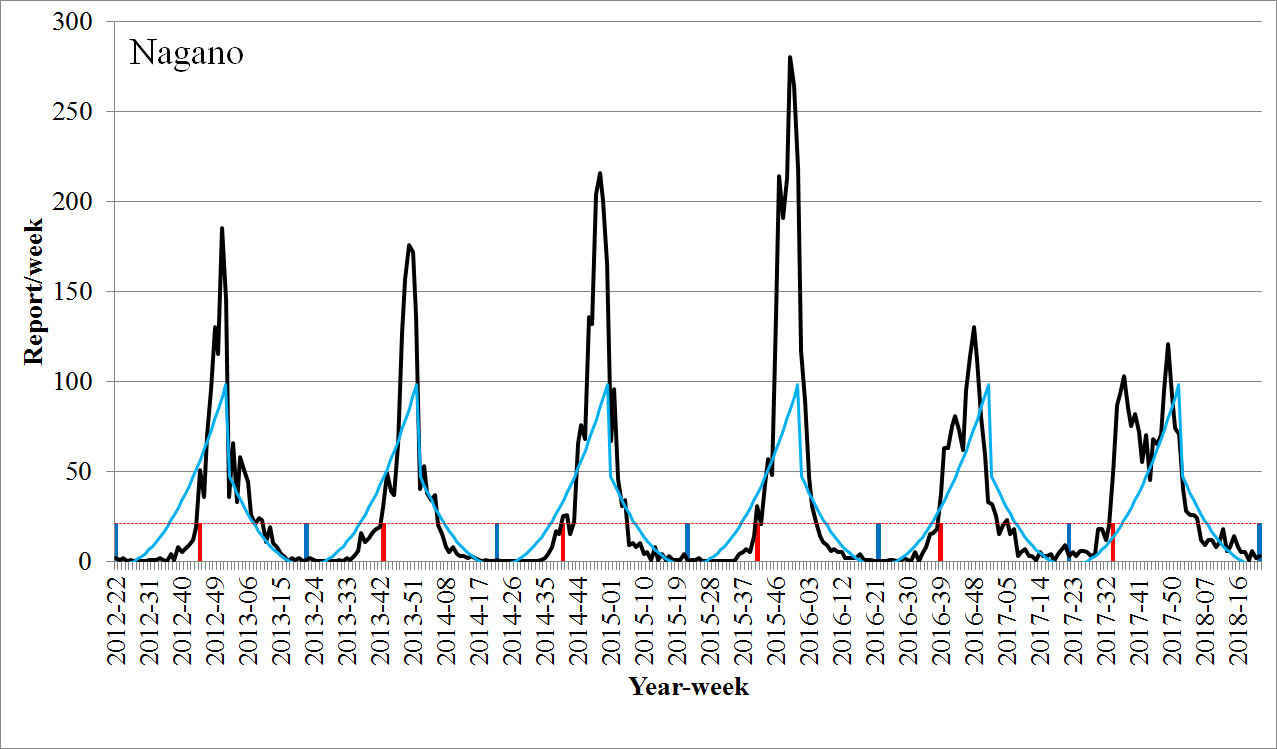

Supplement: Data Sheet 1 — Detection of the start of the epidemic period of respiratory syncytial virus (RSV) infection using the Infectious Diseases Weekly Report surveillance data (2012-2017 seasons) of 46 prefectures. Black line, number of RSV report; light blue line, RSV epidemic cycle; dotted red line, onset line of RSV season; vertical red line, onset week; vertical blue line, trough of epidemic cycle. [file Data_Sheet_1.ZIP › Yamagami et al_Supplementary Figures/Nagano.png]

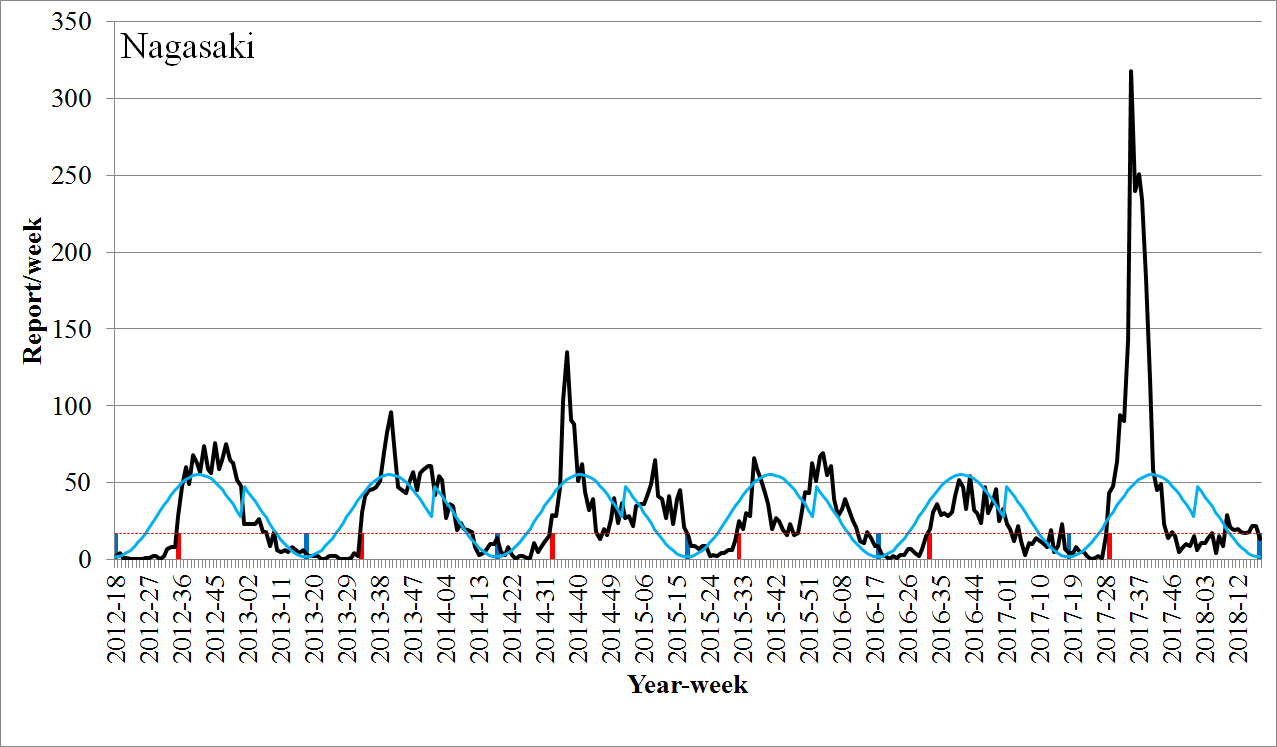

Supplement: Data Sheet 1 — Detection of the start of the epidemic period of respiratory syncytial virus (RSV) infection using the Infectious Diseases Weekly Report surveillance data (2012-2017 seasons) of 46 prefectures. Black line, number of RSV report; light blue line, RSV epidemic cycle; dotted red line, onset line of RSV season; vertical red line, onset week; vertical blue line, trough of epidemic cycle. [file Data_Sheet_1.ZIP › Yamagami et al_Supplementary Figures/Nagasaki.png]

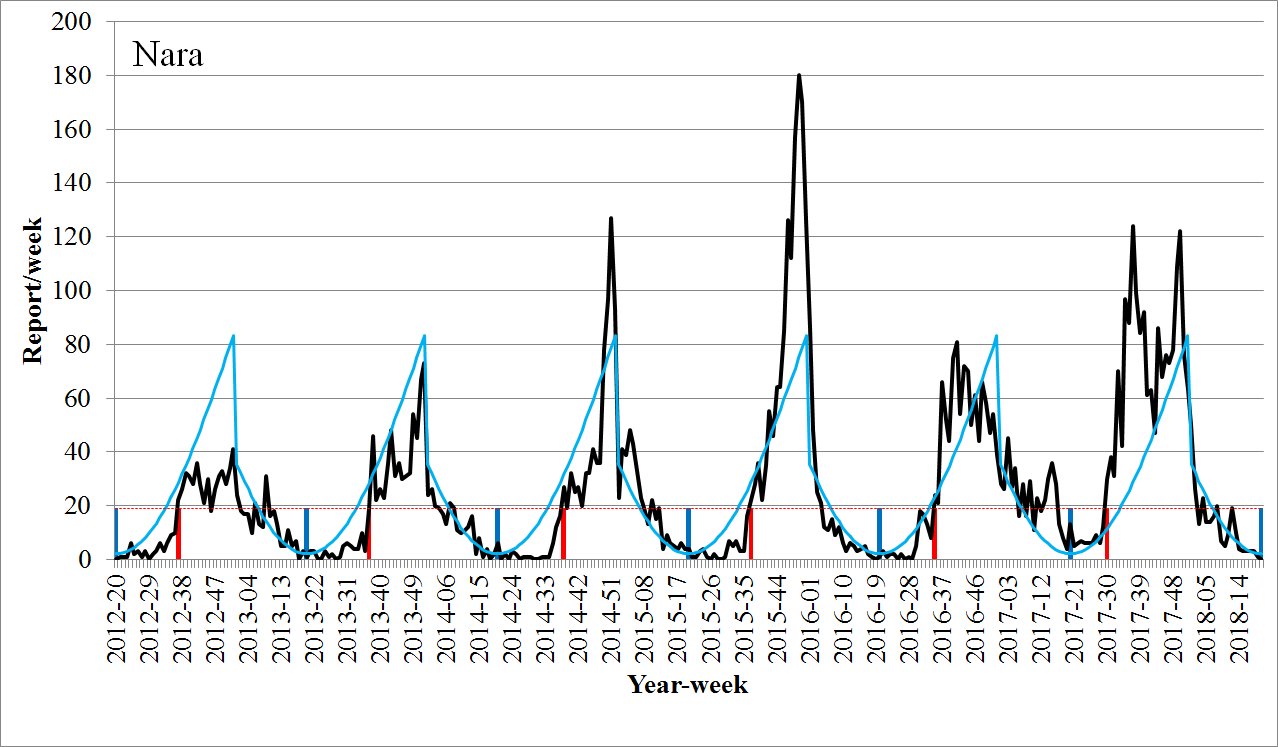

Supplement: Data Sheet 1 — Detection of the start of the epidemic period of respiratory syncytial virus (RSV) infection using the Infectious Diseases Weekly Report surveillance data (2012-2017 seasons) of 46 prefectures. Black line, number of RSV report; light blue line, RSV epidemic cycle; dotted red line, onset line of RSV season; vertical red line, onset week; vertical blue line, trough of epidemic cycle. [file Data_Sheet_1.ZIP › Yamagami et al_Supplementary Figures/Nara.png]

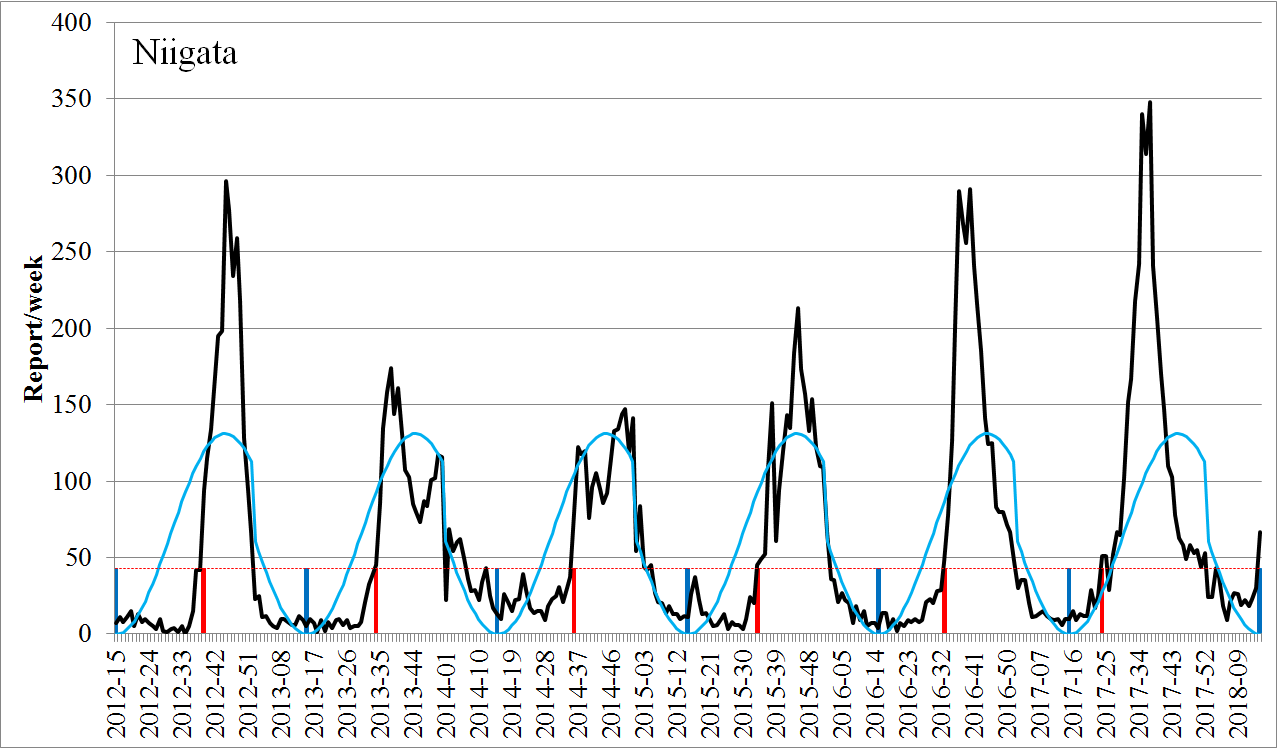

Supplement: Data Sheet 1 — Detection of the start of the epidemic period of respiratory syncytial virus (RSV) infection using the Infectious Diseases Weekly Report surveillance data (2012-2017 seasons) of 46 prefectures. Black line, number of RSV report; light blue line, RSV epidemic cycle; dotted red line, onset line of RSV season; vertical red line, onset week; vertical blue line, trough of epidemic cycle. [file Data_Sheet_1.ZIP › Yamagami et al_Supplementary Figures/Niigata.png]

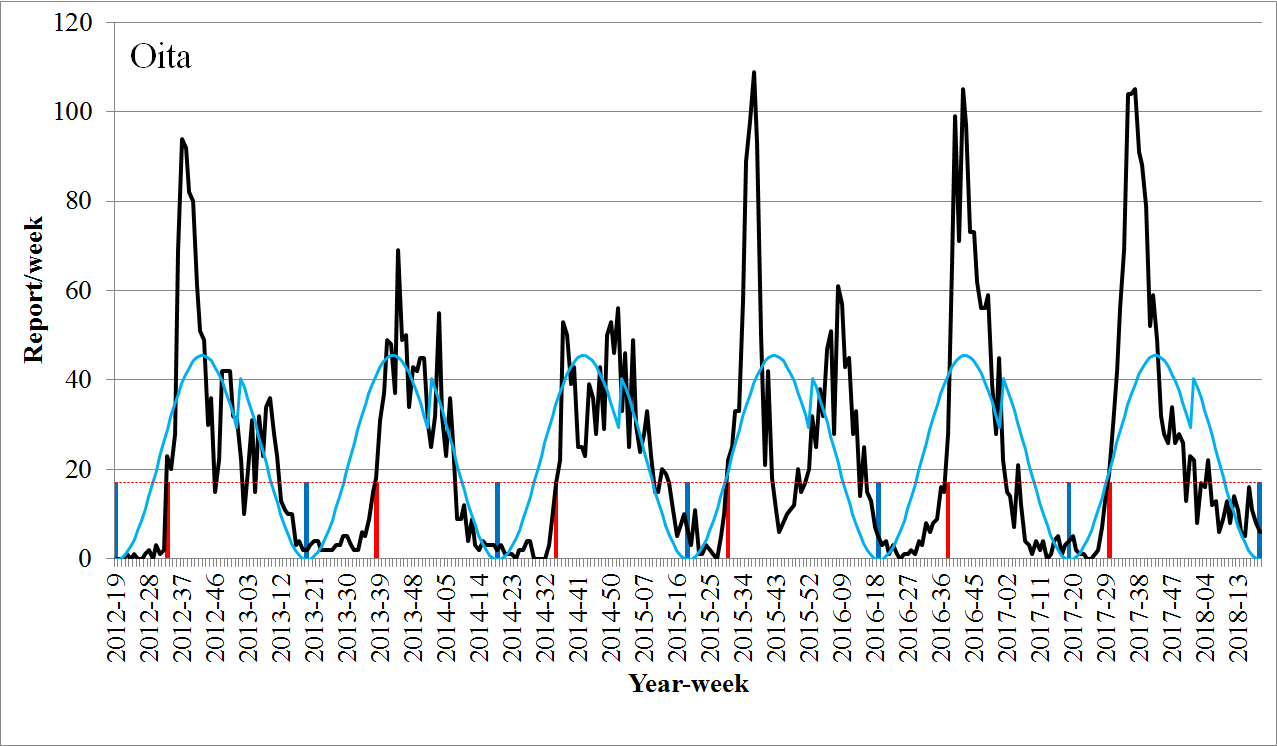

Supplement: Data Sheet 1 — Detection of the start of the epidemic period of respiratory syncytial virus (RSV) infection using the Infectious Diseases Weekly Report surveillance data (2012-2017 seasons) of 46 prefectures. Black line, number of RSV report; light blue line, RSV epidemic cycle; dotted red line, onset line of RSV season; vertical red line, onset week; vertical blue line, trough of epidemic cycle. [file Data_Sheet_1.ZIP › Yamagami et al_Supplementary Figures/Oita.png]

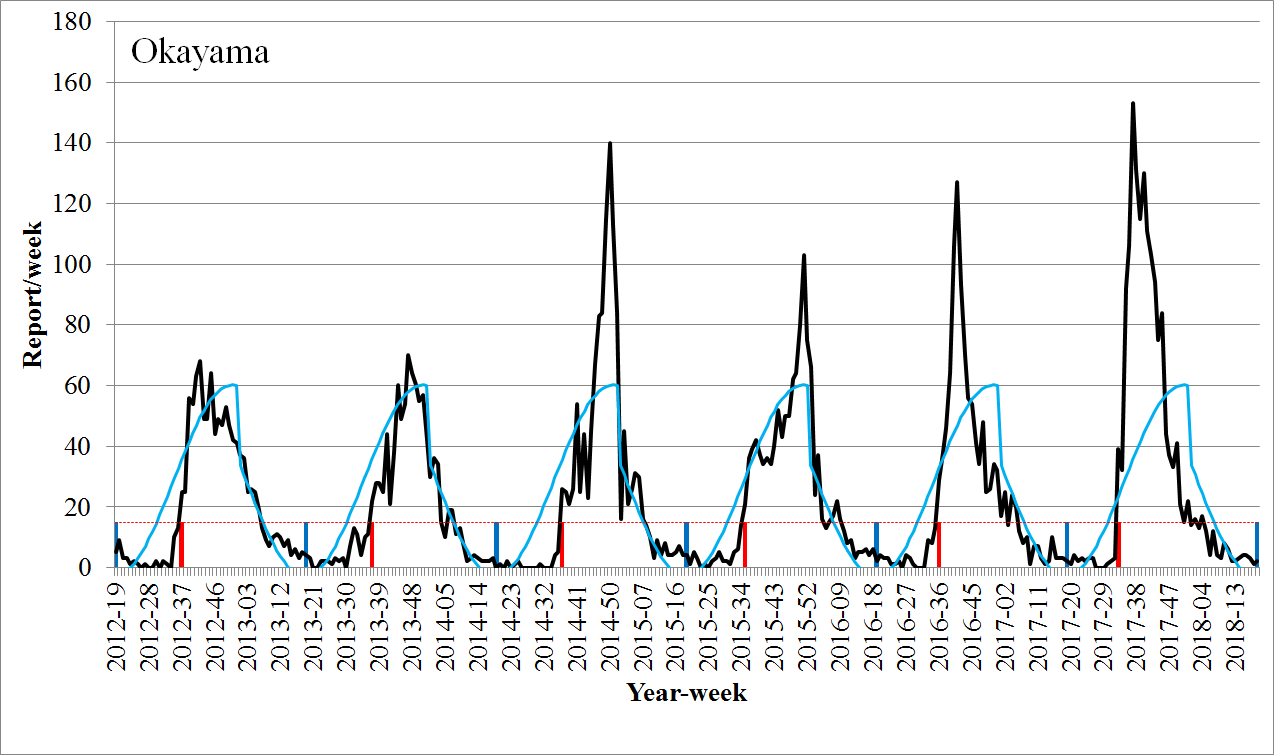

Supplement: Data Sheet 1 — Detection of the start of the epidemic period of respiratory syncytial virus (RSV) infection using the Infectious Diseases Weekly Report surveillance data (2012-2017 seasons) of 46 prefectures. Black line, number of RSV report; light blue line, RSV epidemic cycle; dotted red line, onset line of RSV season; vertical red line, onset week; vertical blue line, trough of epidemic cycle. [file Data_Sheet_1.ZIP › Yamagami et al_Supplementary Figures/Okayama.png]

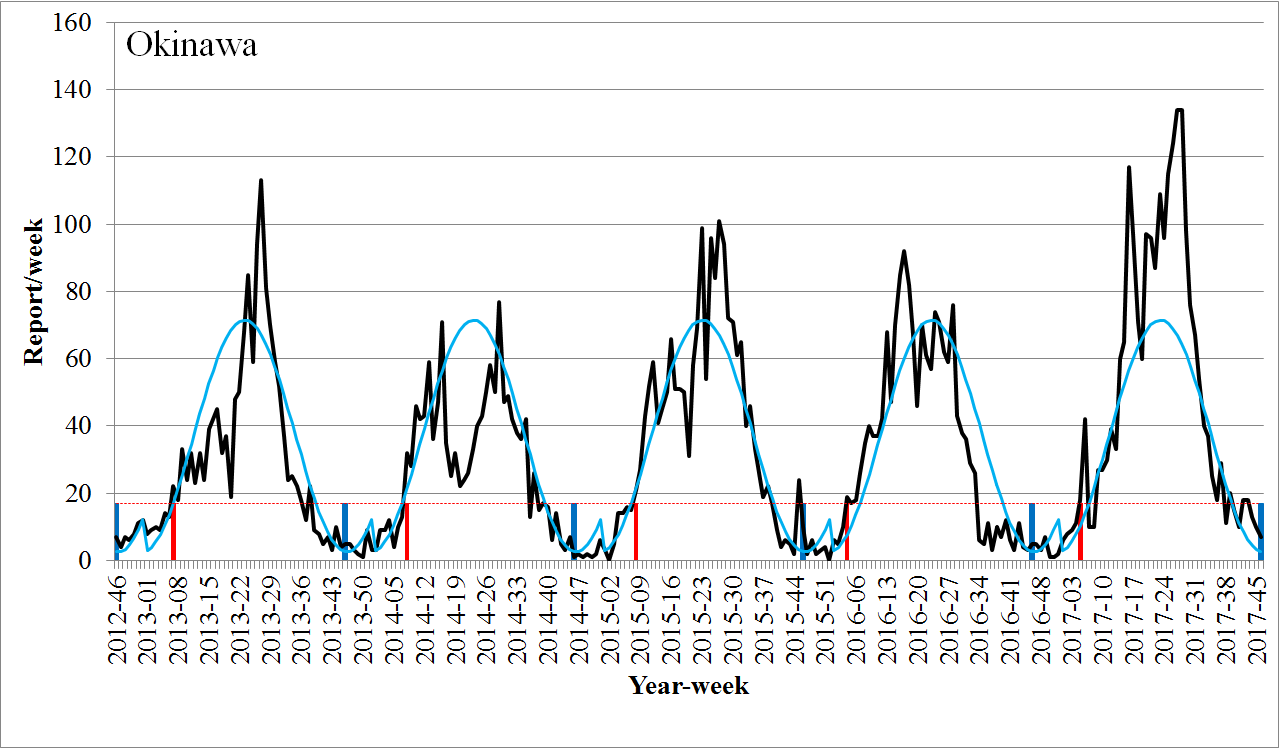

Supplement: Data Sheet 1 — Detection of the start of the epidemic period of respiratory syncytial virus (RSV) infection using the Infectious Diseases Weekly Report surveillance data (2012-2017 seasons) of 46 prefectures. Black line, number of RSV report; light blue line, RSV epidemic cycle; dotted red line, onset line of RSV season; vertical red line, onset week; vertical blue line, trough of epidemic cycle. [file Data_Sheet_1.ZIP › Yamagami et al_Supplementary Figures/Okinawa.png]

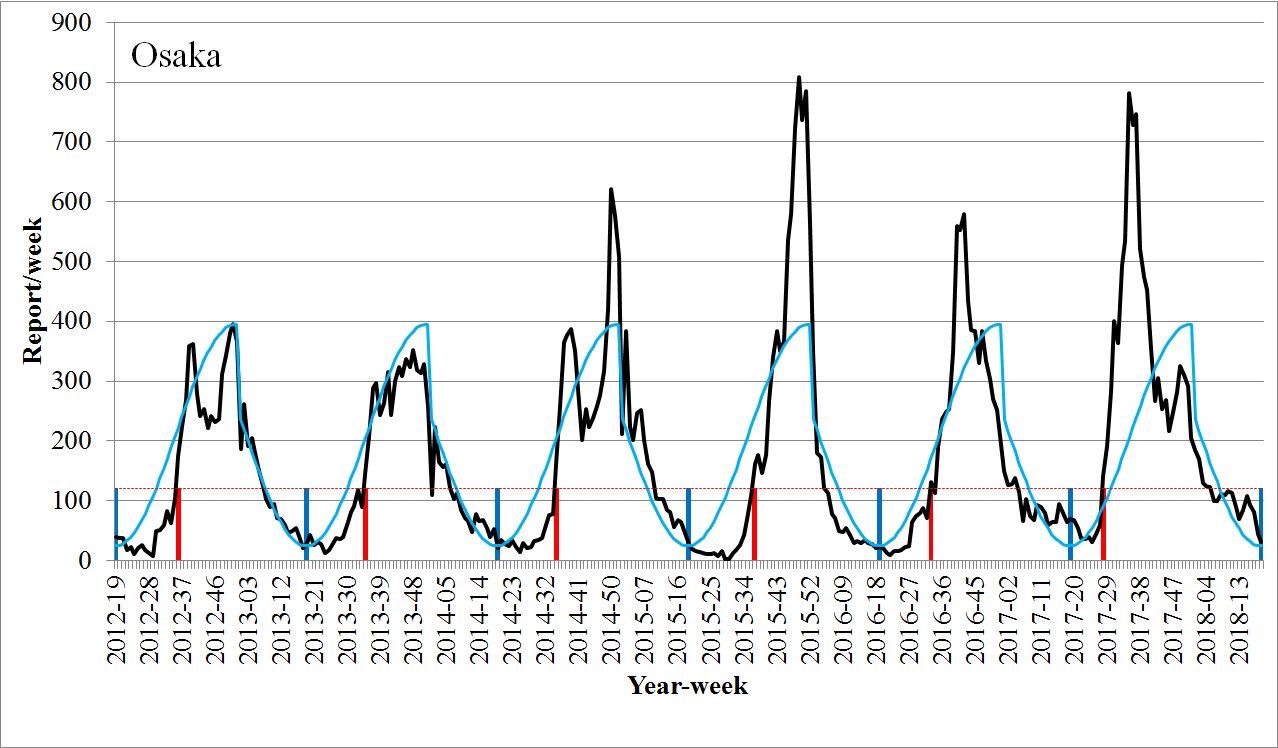

Supplement: Data Sheet 1 — Detection of the start of the epidemic period of respiratory syncytial virus (RSV) infection using the Infectious Diseases Weekly Report surveillance data (2012-2017 seasons) of 46 prefectures. Black line, number of RSV report; light blue line, RSV epidemic cycle; dotted red line, onset line of RSV season; vertical red line, onset week; vertical blue line, trough of epidemic cycle. [file Data_Sheet_1.ZIP › Yamagami et al_Supplementary Figures/Osaka.png]

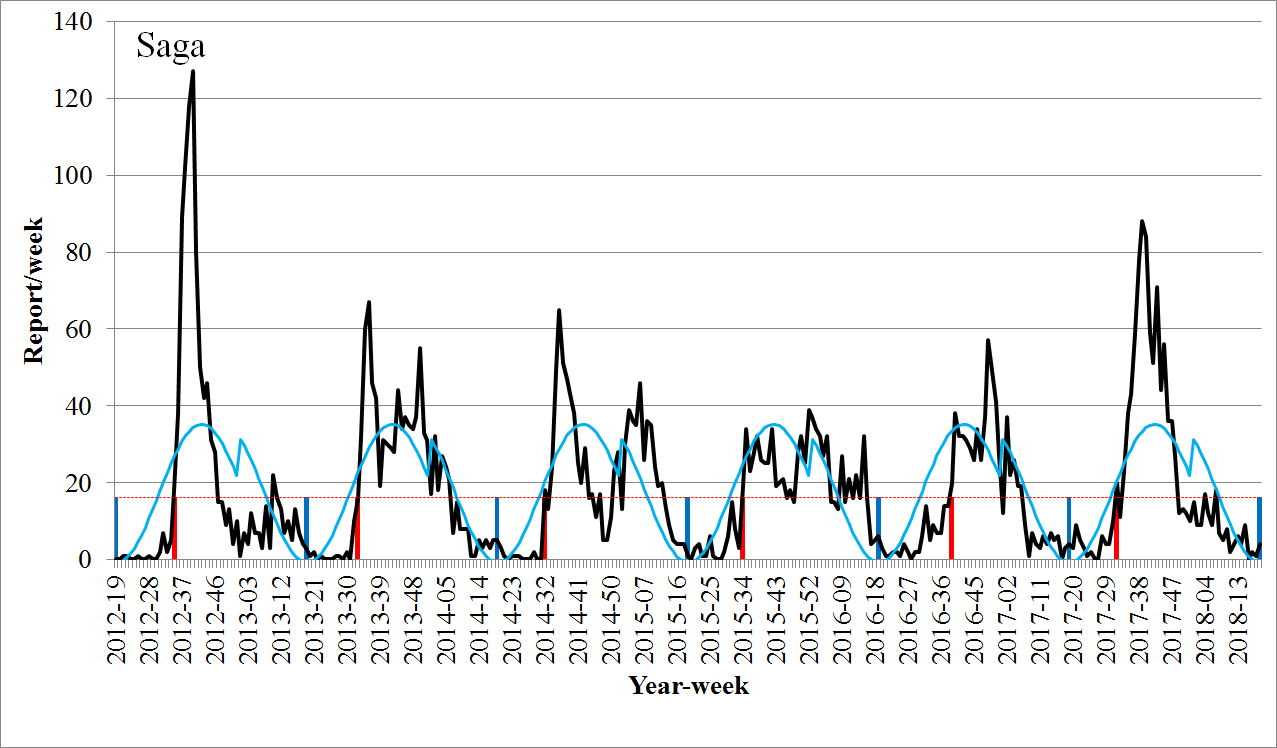

Supplement: Data Sheet 1 — Detection of the start of the epidemic period of respiratory syncytial virus (RSV) infection using the Infectious Diseases Weekly Report surveillance data (2012-2017 seasons) of 46 prefectures. Black line, number of RSV report; light blue line, RSV epidemic cycle; dotted red line, onset line of RSV season; vertical red line, onset week; vertical blue line, trough of epidemic cycle. [file Data_Sheet_1.ZIP › Yamagami et al_Supplementary Figures/Saga.png]

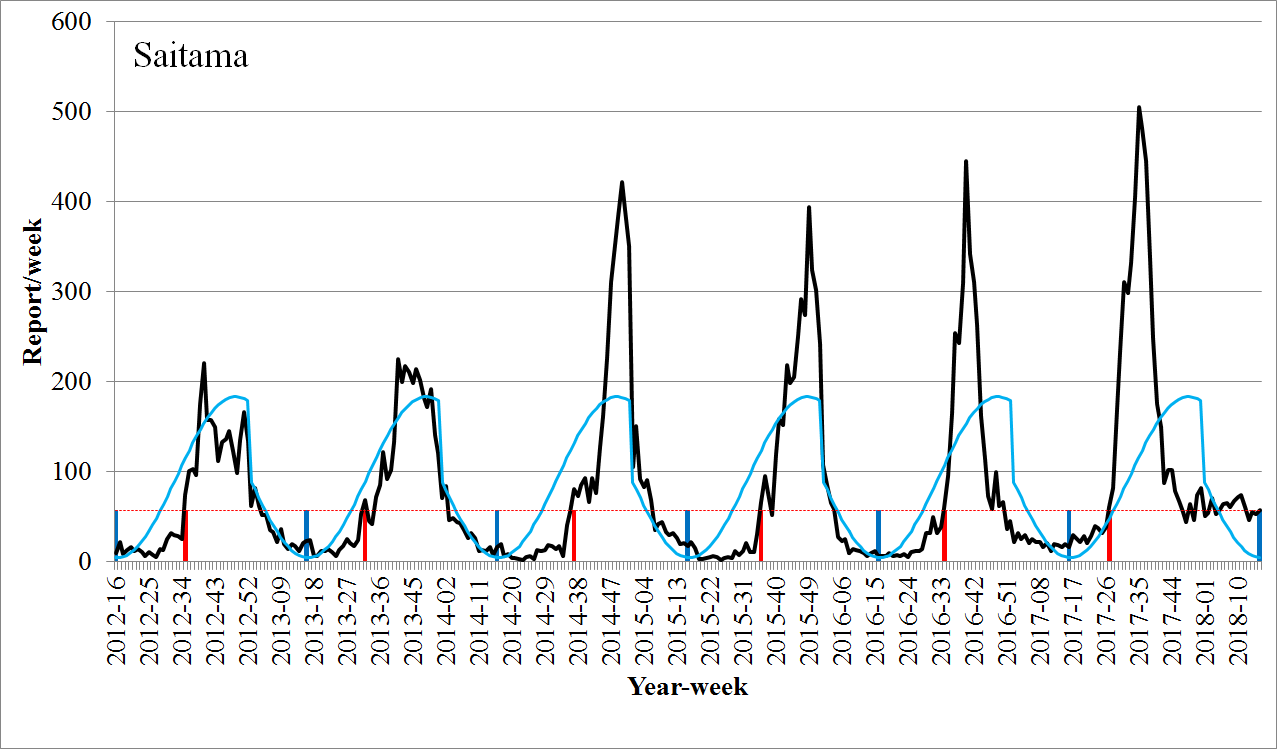

Supplement: Data Sheet 1 — Detection of the start of the epidemic period of respiratory syncytial virus (RSV) infection using the Infectious Diseases Weekly Report surveillance data (2012-2017 seasons) of 46 prefectures. Black line, number of RSV report; light blue line, RSV epidemic cycle; dotted red line, onset line of RSV season; vertical red line, onset week; vertical blue line, trough of epidemic cycle. [file Data_Sheet_1.ZIP › Yamagami et al_Supplementary Figures/Saitama.png]

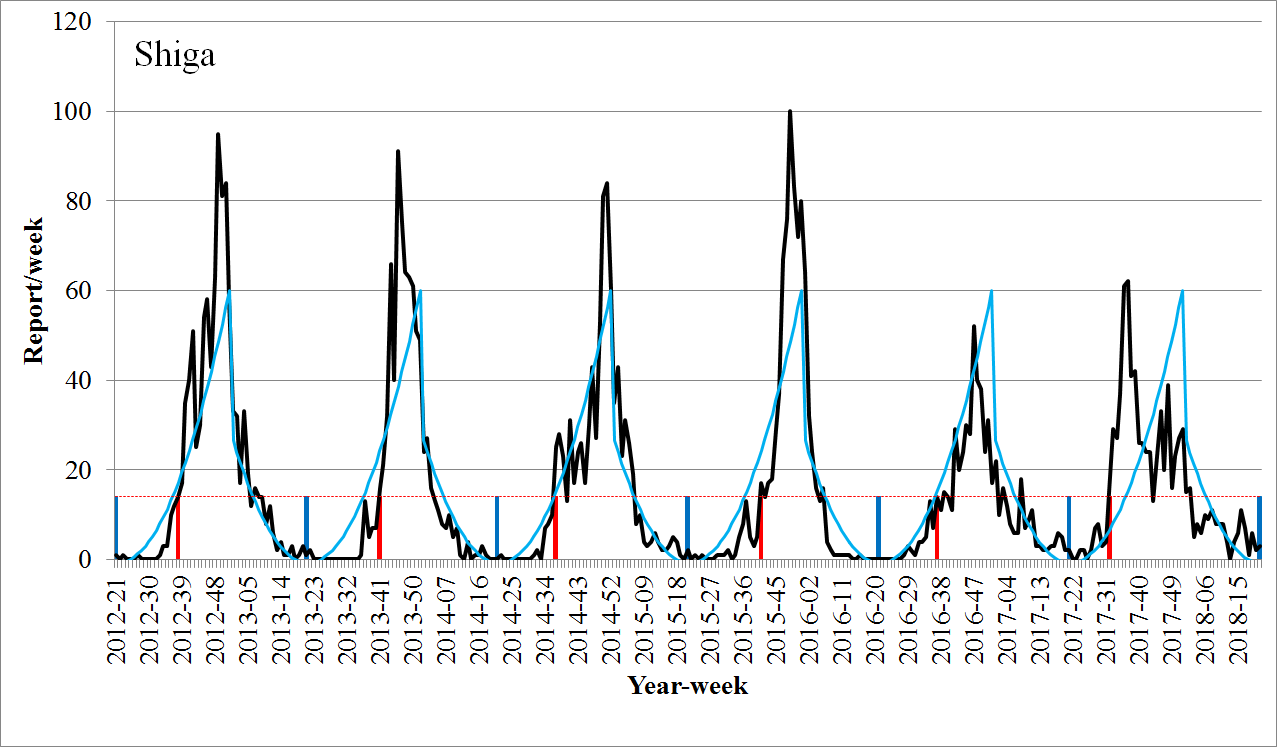

Supplement: Data Sheet 1 — Detection of the start of the epidemic period of respiratory syncytial virus (RSV) infection using the Infectious Diseases Weekly Report surveillance data (2012-2017 seasons) of 46 prefectures. Black line, number of RSV report; light blue line, RSV epidemic cycle; dotted red line, onset line of RSV season; vertical red line, onset week; vertical blue line, trough of epidemic cycle. [file Data_Sheet_1.ZIP › Yamagami et al_Supplementary Figures/Shiga.png]

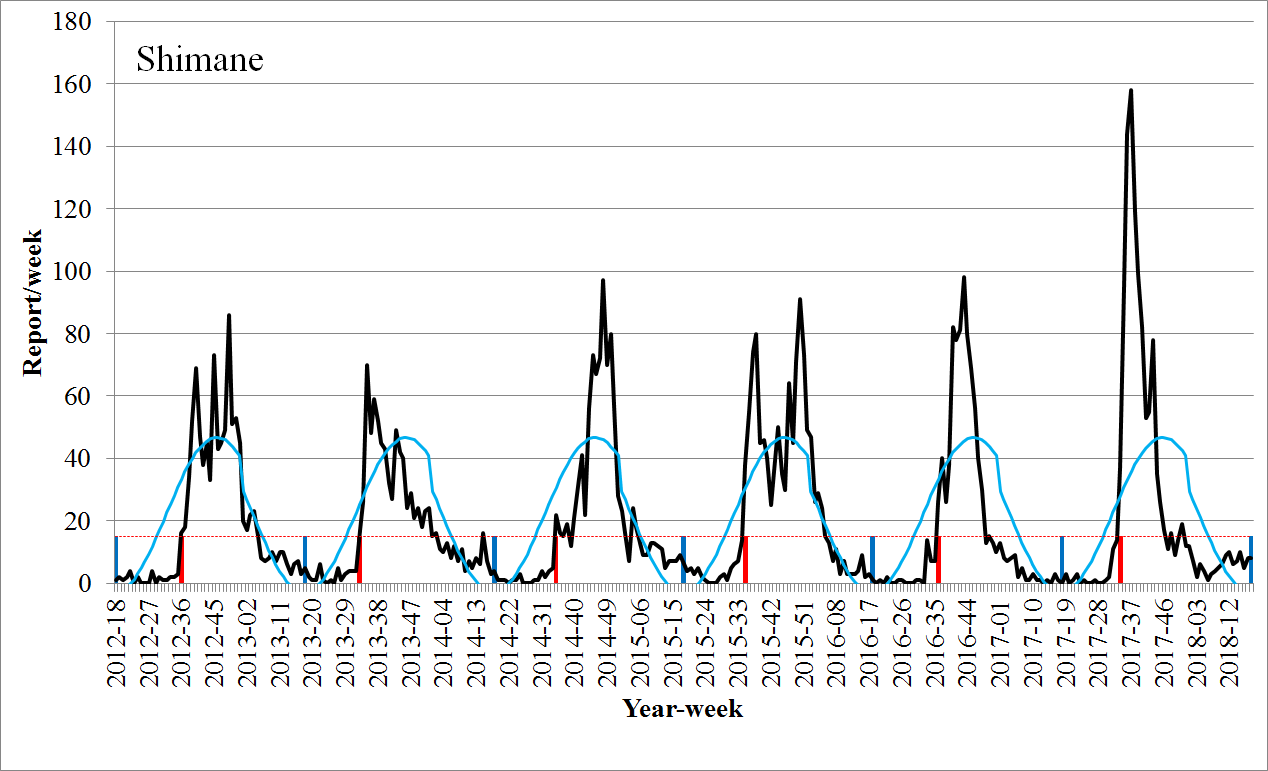

Supplement: Data Sheet 1 — Detection of the start of the epidemic period of respiratory syncytial virus (RSV) infection using the Infectious Diseases Weekly Report surveillance data (2012-2017 seasons) of 46 prefectures. Black line, number of RSV report; light blue line, RSV epidemic cycle; dotted red line, onset line of RSV season; vertical red line, onset week; vertical blue line, trough of epidemic cycle. [file Data_Sheet_1.ZIP › Yamagami et al_Supplementary Figures/Shimane.png]

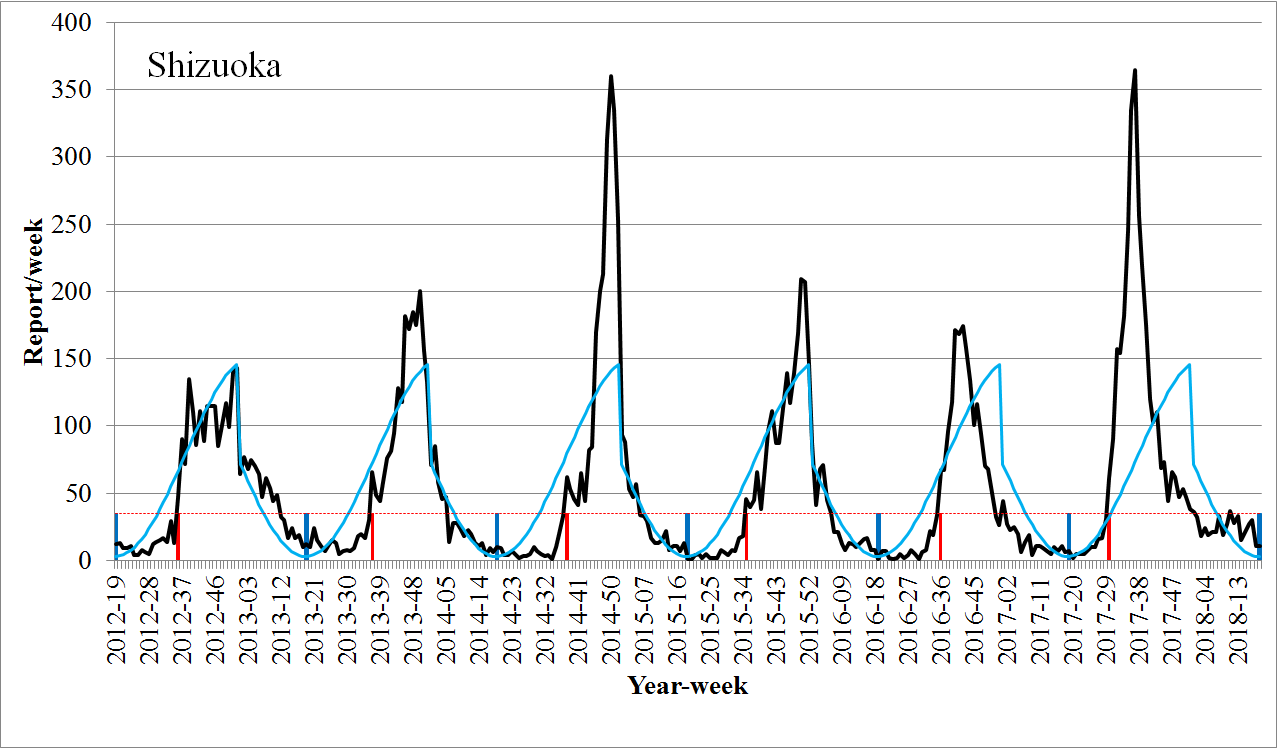

Supplement: Data Sheet 1 — Detection of the start of the epidemic period of respiratory syncytial virus (RSV) infection using the Infectious Diseases Weekly Report surveillance data (2012-2017 seasons) of 46 prefectures. Black line, number of RSV report; light blue line, RSV epidemic cycle; dotted red line, onset line of RSV season; vertical red line, onset week; vertical blue line, trough of epidemic cycle. [file Data_Sheet_1.ZIP › Yamagami et al_Supplementary Figures/Shizuoka.png]

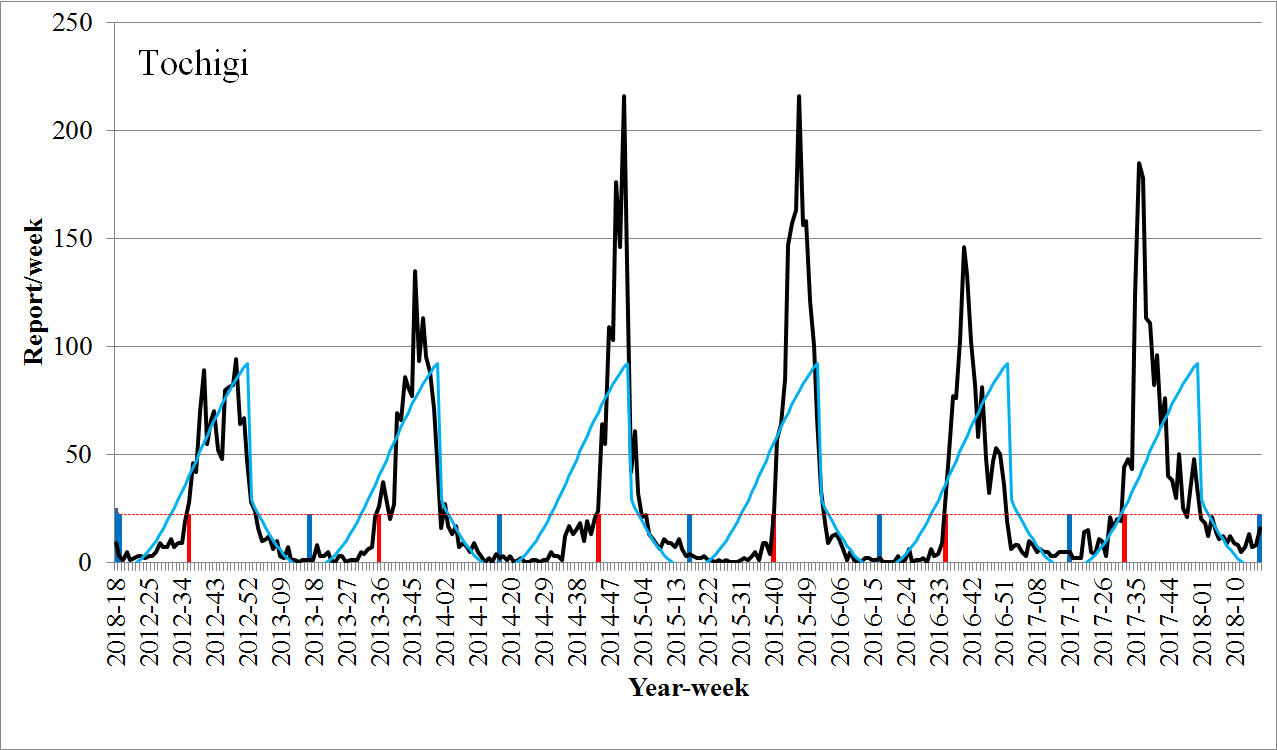

Supplement: Data Sheet 1 — Detection of the start of the epidemic period of respiratory syncytial virus (RSV) infection using the Infectious Diseases Weekly Report surveillance data (2012-2017 seasons) of 46 prefectures. Black line, number of RSV report; light blue line, RSV epidemic cycle; dotted red line, onset line of RSV season; vertical red line, onset week; vertical blue line, trough of epidemic cycle. [file Data_Sheet_1.ZIP › Yamagami et al_Supplementary Figures/Tochigi.png]

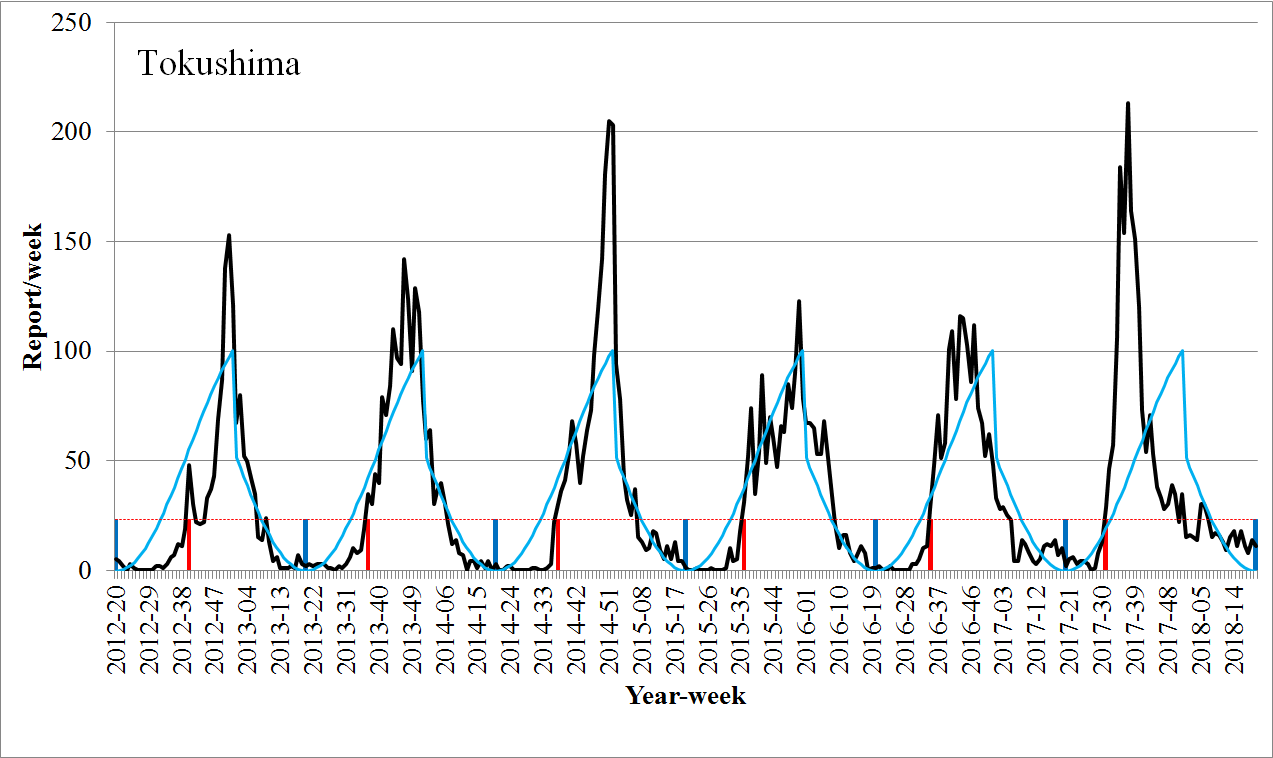

Supplement: Data Sheet 1 — Detection of the start of the epidemic period of respiratory syncytial virus (RSV) infection using the Infectious Diseases Weekly Report surveillance data (2012-2017 seasons) of 46 prefectures. Black line, number of RSV report; light blue line, RSV epidemic cycle; dotted red line, onset line of RSV season; vertical red line, onset week; vertical blue line, trough of epidemic cycle. [file Data_Sheet_1.ZIP › Yamagami et al_Supplementary Figures/Tokushima.png]

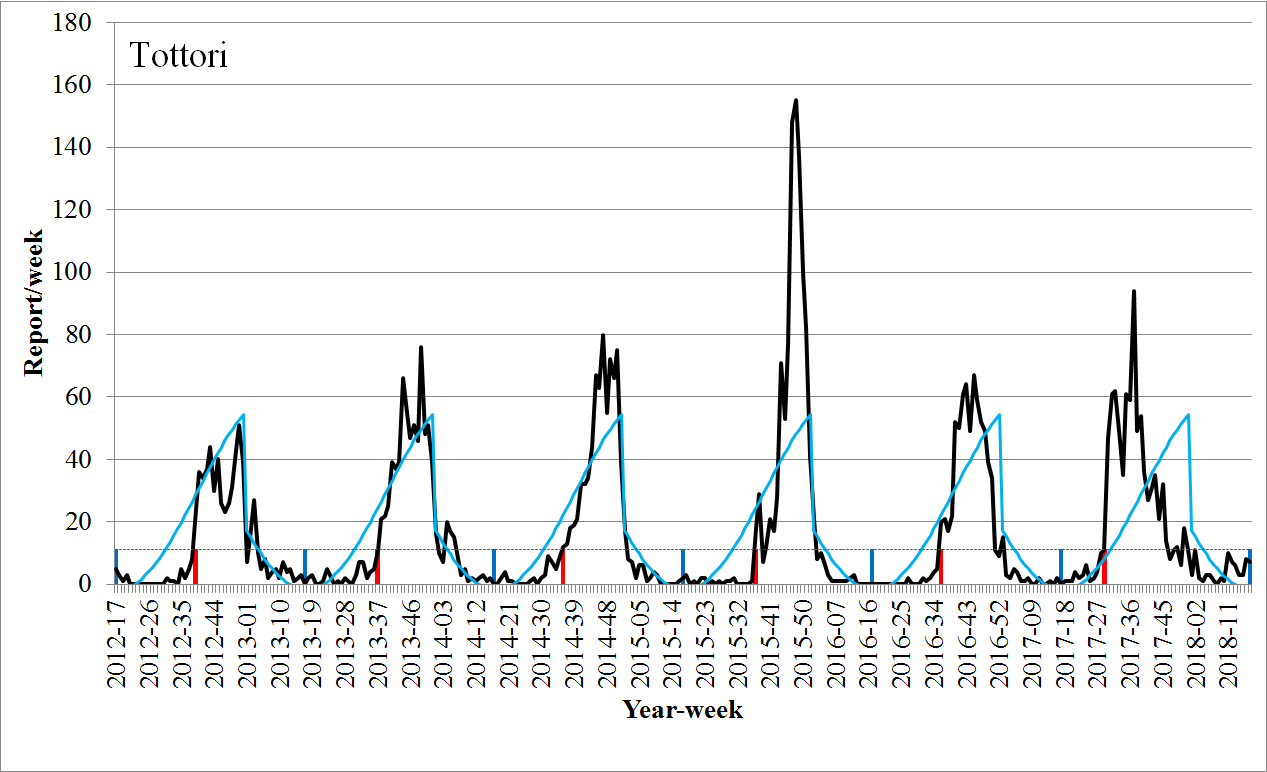

Supplement: Data Sheet 1 — Detection of the start of the epidemic period of respiratory syncytial virus (RSV) infection using the Infectious Diseases Weekly Report surveillance data (2012-2017 seasons) of 46 prefectures. Black line, number of RSV report; light blue line, RSV epidemic cycle; dotted red line, onset line of RSV season; vertical red line, onset week; vertical blue line, trough of epidemic cycle. [file Data_Sheet_1.ZIP › Yamagami et al_Supplementary Figures/Tottori.png]

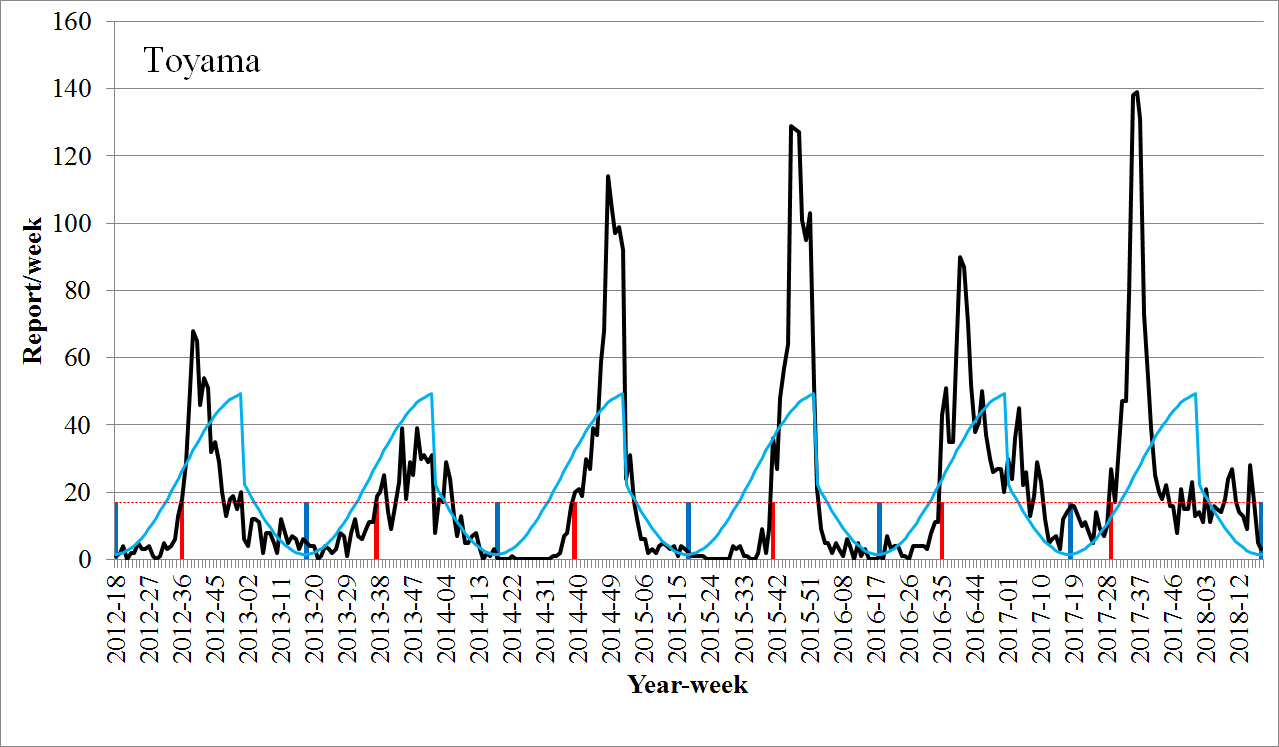

Supplement: Data Sheet 1 — Detection of the start of the epidemic period of respiratory syncytial virus (RSV) infection using the Infectious Diseases Weekly Report surveillance data (2012-2017 seasons) of 46 prefectures. Black line, number of RSV report; light blue line, RSV epidemic cycle; dotted red line, onset line of RSV season; vertical red line, onset week; vertical blue line, trough of epidemic cycle. [file Data_Sheet_1.ZIP › Yamagami et al_Supplementary Figures/Toyama.png]

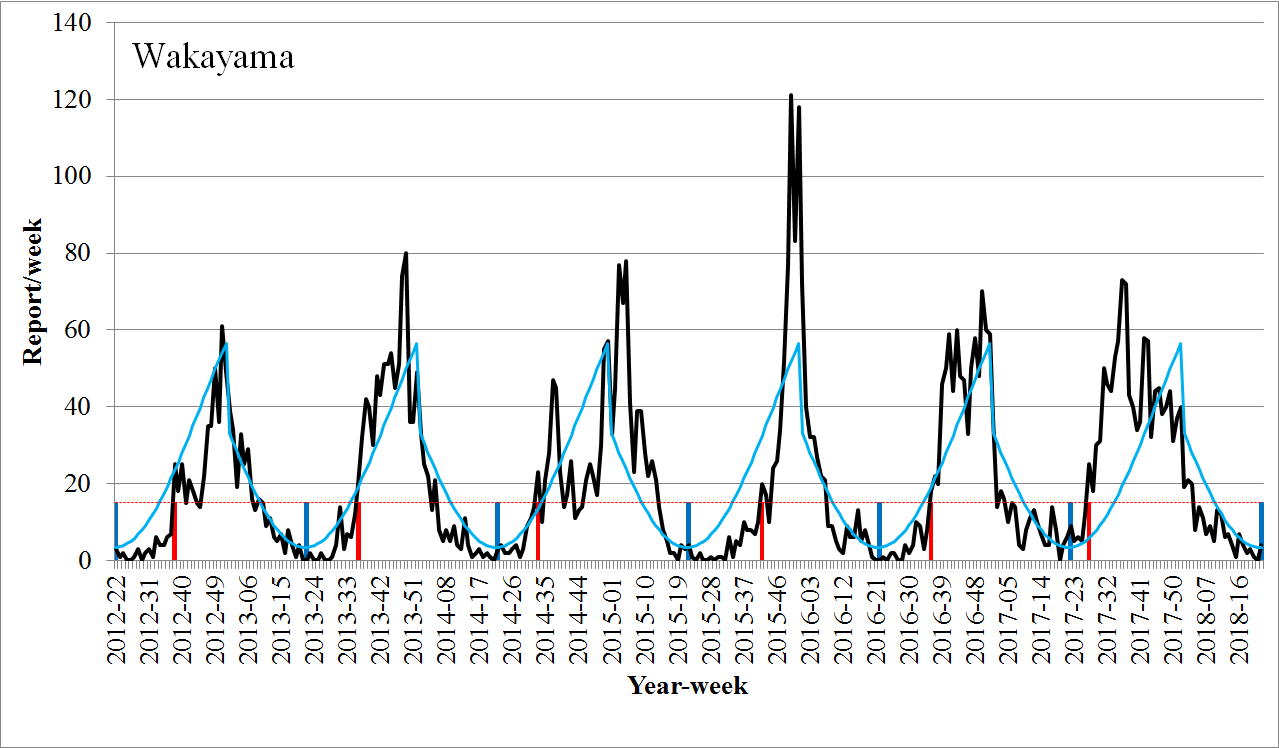

Supplement: Data Sheet 1 — Detection of the start of the epidemic period of respiratory syncytial virus (RSV) infection using the Infectious Diseases Weekly Report surveillance data (2012-2017 seasons) of 46 prefectures. Black line, number of RSV report; light blue line, RSV epidemic cycle; dotted red line, onset line of RSV season; vertical red line, onset week; vertical blue line, trough of epidemic cycle. [file Data_Sheet_1.ZIP › Yamagami et al_Supplementary Figures/Wakayama.png]

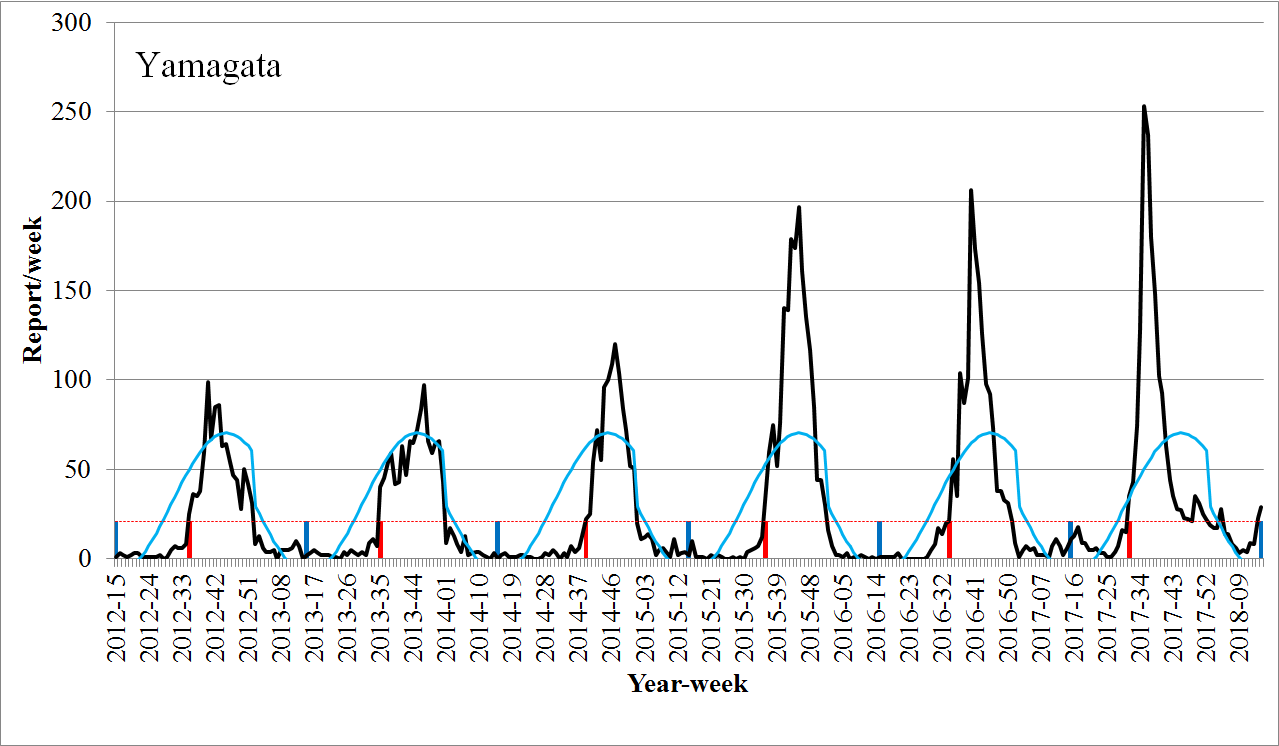

Supplement: Data Sheet 1 — Detection of the start of the epidemic period of respiratory syncytial virus (RSV) infection using the Infectious Diseases Weekly Report surveillance data (2012-2017 seasons) of 46 prefectures. Black line, number of RSV report; light blue line, RSV epidemic cycle; dotted red line, onset line of RSV season; vertical red line, onset week; vertical blue line, trough of epidemic cycle. [file Data_Sheet_1.ZIP › Yamagami et al_Supplementary Figures/Yamagata.png]

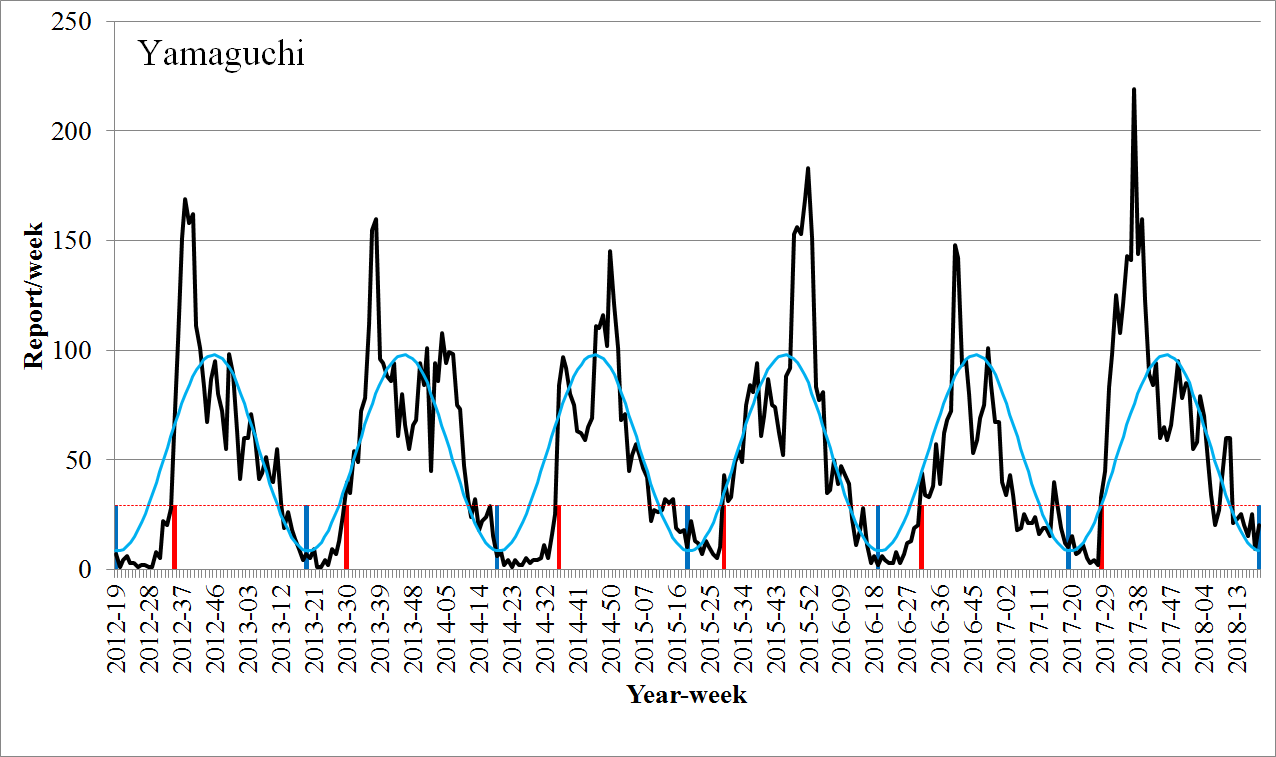

Supplement: Data Sheet 1 — Detection of the start of the epidemic period of respiratory syncytial virus (RSV) infection using the Infectious Diseases Weekly Report surveillance data (2012-2017 seasons) of 46 prefectures. Black line, number of RSV report; light blue line, RSV epidemic cycle; dotted red line, onset line of RSV season; vertical red line, onset week; vertical blue line, trough of epidemic cycle. [file Data_Sheet_1.ZIP › Yamagami et al_Supplementary Figures/Yamaguchi.png]

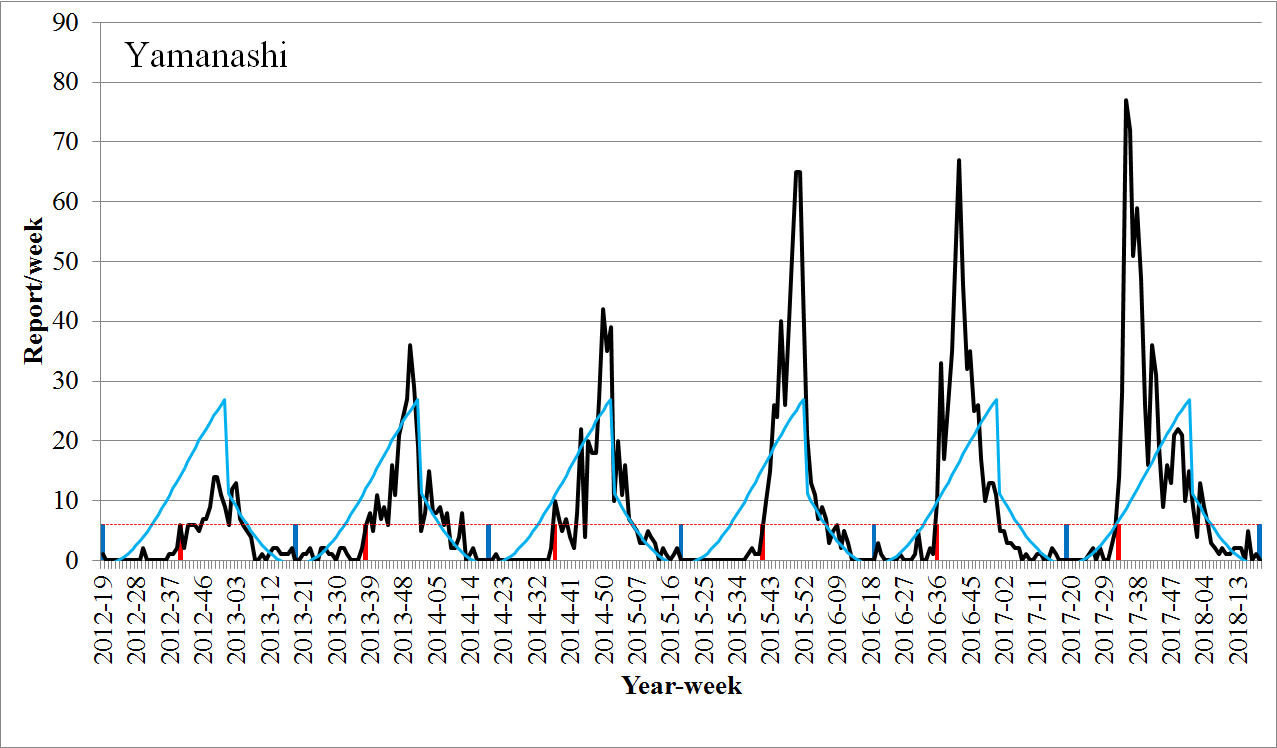

Supplement: Data Sheet 1 — Detection of the start of the epidemic period of respiratory syncytial virus (RSV) infection using the Infectious Diseases Weekly Report surveillance data (2012-2017 seasons) of 46 prefectures. Black line, number of RSV report; light blue line, RSV epidemic cycle; dotted red line, onset line of RSV season; vertical red line, onset week; vertical blue line, trough of epidemic cycle. [file Data_Sheet_1.ZIP › Yamagami et al_Supplementary Figures/Yamanashi.png]
